# Gene editing enables non-invasive *in vivo* PET imaging of human induced pluripotent stem cell-derived liver bud organoids

**DOI:** 10.1016/j.omtm.2025.101406

**Published:** 2025-01-07

**Authors:** Candice Ashmore-Harris, Hiroaki Ayabe, Emi Yoshizawa, Tetsu Arisawa, Yuuki Takada, Takanori Takebe, Gilbert O. Fruhwirth

**Affiliations:** 1 Centre for Regenerative Medicine, Institute for Regeneration and Repair, The University of Edinburgh, Edinburgh BioQuarter, 5 Little France Drive, Edinburgh EH16 4UU, UK; 2 Imaging Therapies and Cancer Group, Comprehensive Cancer Centre, School of Cancer and Pharmaceutical Sciences, King’s College London, Guy’s Cancer Centre, London SE1 1UL, UK; 3 Department of Regenerative Medicine, Graduate School of Medicine, Yokohama City University, Yokohama, 3-9 Fukuura, Kanazawa-ku, Yokohama, Kanagawa 236-0004, Japan; 4 Department of Pediatrics, University of Texas Southwestern Medical Center, 5323 Harry Hines Boulevard, Dallas, TX 75390, USA; 5 Department of Physiology, Graduate School of Medicine, Yokohama City University, Yokohama, 3-9 Fukuura, Kanazawa-ku, Yokohama, Kanagawa 236-0004, Japan; 6 Center for Stem Cell & Organoid Medicine (CuSTOM), Division of Gastroenterology, Hepatology and Nutrition & Division of Developmental Biology, Cincinnati Children’s Hospital Medical Center, 3333 Burnet Avenue, Cincinnati, OH 45229-3039, USA; 7 Premium Research Institute for Human Metaverse Medicine (WPI-PRIMe), and Division of Stem Cell and Organoid Medicine, Osaka University, Suita, Osaka 565-0871, Japan

**Keywords:** cell tracking, hepatocyte-like cells, hiPSC, reporter gene imaging, sodium iodide symporter, gene editing, PET, regenerative medicine

## Abstract

Human induced pluripotent stem cell (hiPSC)-derived liver cell therapies such as hepatocyte-like cells and liver organoids could provide unlimited therapeutic cells for clinical transplantation, but an inadequate understanding of their *in vivo* fate impedes translation. Whole body *in vivo* imaging could enable monitoring of transplanted cell survival and/or expansion non-invasively over time, permitting robust comparisons between emerging therapies to identify those most effective. The human sodium iodide symporter (hNIS) is a radionuclide reporter gene facilitating whole body *in vivo* cell tracking by positron emission tomography (PET). We gene-edited a clinical Good Manufacturing Practice-compliant hiPSC line at the AAVS1 safe harbor locus enabling constitutive expression of a hNIS-monomeric(m)GFP fusion reporter in hiPSCs and their differentiated progeny. We confirmed reporter integration did not impact pluripotency or differentiation capacity, and radiotracer uptake capacity was retained post-differentiation. *In vivo* trackable liver bud (LB) organoids were generated from traceable hNIS fused to monomeric GFP (hNIS-mGFP)-hiPSCs and transplanted into healthy and liver-injured mice. LB were imaged quantitatively by 18FBF_4_^−^-PET with imaging results confirmed histologically. We report, for the first time, hNIS-mGFP-hiPSC progeny retain differentiated function and PET trackability *in vivo* using LB. *In vivo* monitoring could accelerate regenerative cell therapy development by identifying efficacious candidate cells, successful engraftment/survival strategies and addressing safety concerns.

## Graphical abstract

**Figure:**
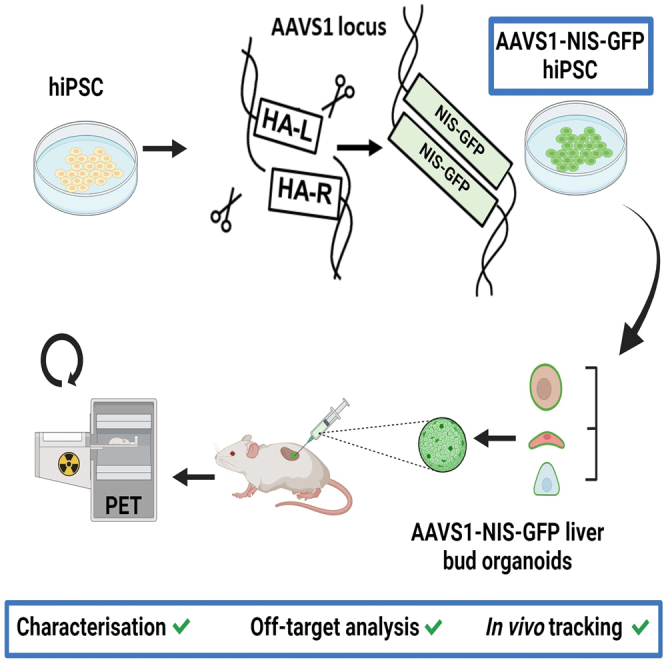

## Teaser

hiPSCs represent a promising route to cell therapy products. Ashmore-Harris and colleagues gene-edited cGMP-compliant hiPSCs with an imaging reporter, characterized them, and thereby enabled non-invasive *in vivo* PET-tracking of liver-bud organoids. This approach boosts hiPSC therapy development and can enable future non-invasive clinical therapy monitoring.

## Introduction

Mortality from liver disease continues to rise despite extensive research.^1^ Orthotopic liver transplantation (OLT) remains the only definitive treatment for acute, end-stage, and metabolic liver disease, yet patient need for liver transplants consistently exceeds the availability of donor organs. As a result, many patients die each year while on the waiting list for a suitable transplant, or are removed from it due to deterioration of their condition.^2^^,^^3^^,^^4^ Consequently, alternatives to OLT are urgently needed to expand treatment capacity. Hepatocyte transplants (HTxs)^5^^,^^6^ are currently a promising alternative to OLT, with more than 100 patients already treated worldwide.^6^ Many HTx patients have been successfully bridged to OLT or made a full recovery without the need for OLT.^7^ Although promising, HTx has notable caveats, including variable cell engraftment and often only transient graft survival. This is partly explained by limited primary hepatocyte quality. While hepatocytes comprise up to 80% of the liver volume and cell population,^8^ sourcing is constrained to donor livers that were rejected for OLT; thus, the cells are mostly isolated from aged donors, livers with steatosis, livers with an aberrant anatomy, or those having undergone prolonged ischemia, all of which negatively impact on hepatocyte quality.^9^ The self-renewal capacity of human pluripotent stem cells (hPSCs) and their ability to differentiate into a range of prospective cell types, including hepatocyte-like cells (HLCs), hepatic progenitors, and liver organoids, has led to widespread investigation into their potential to produce an unlimited supply of high-quality liver cell therapies to act as an alternative source to primary hepatocytes. However, considerable variations in hPSC differentiation protocols,^10^ transplantation niches (e.g., ectopic or orthotopic sites, splenic or intraportal delivery), and optimal cell delivery formats (single cell suspensions, organoids, hepatocyte sheets, etc.) make identifying which emerging therapies may be the most promising upon translation difficult. Additionally, like primary cell HTx, hiPSC-derived liver cell therapies suffer from poor initial engraftment and graft transience (assumed to be due to immune mediated cell destruction over time). Systematic investigations aimed at tackling therapeutic challenges, e.g., approaches that enable the quantification of engraftment and cell survival in real time, longitudinally in the same subject are essential to address these translational bottlenecks.

Whole body *in vivo* imaging can facilitate direct monitoring of cell survival and/or expansion non-invasively, thereby enabling comparisons and benchmarking between various emerging therapeutic approaches. Positron emission tomography (PET) is currently best placed for this purpose, offering the highest sensitivity of the available clinically compatible imaging modalities as well as absolute signal quantification and 3D data at depth.^11^ To generate contrast, cells of interest need to be labeled, whereby indirect cell labeling exploiting a radionuclide reporter gene is best suited for this application.^12^ To ensure immunocompatibility, a host reporter gene is required. The human sodium iodide symporter (hNIS) is a very promising candidate for this purpose, because liver tissues are free from any endogenous hNIS expression and its corresponding radionuclide imaging probes, e.g., F-18-tetrafluoroborate (18FBF_4_^−^, for PET) and Tc-99m-pertechnetate ([^99m^Tc]TcO_4_^−^, for single photon emission computed tomography [SPECT]) are excreted exclusively via the renal system.^11^ hNIS-afforded *in vivo* cell tracking has been successfully demonstrated for diverse cell types including immune cells,^13^^,^^14^ cancer cells,^15^^,^^16^^,^^17^ and various stem cells,^11^^,^^18^^,^^19^ with homologous animal NIS variants also used for tracking myoblasts^20^^,^^21^ and hPSCs.^22^ We previously demonstrated that lentiviral transduction-afforded expression of hNIS fused to monomeric GFP (hNIS-mGFP) enabled human induced pluripotent stem cell (hiPSC)-derived HLC tracking *in vivo* by SPECT/CT imaging with no detectable impact on the hepatic phenotype.^23^ This dual reporter approach also enables linked whole body imaging data to *ex vivo* microscopy; however, this study was limited in the ability to track only HLCs, as transduction of progenitor cells resulted in downstream reporter silencing.

Furthermore, adopting a virus-based approach for producing sufficient transgenic HLCs for large-scale studies, e.g., transplant optimization studies, is very resource and labor intensive particularly as substantial quantities of high-titer lentiviral particles are required to transduce sufficient differentiated HLCs; bulk viral vector production remains a major constraint in cell therapy engineering.^24^ Moreover, the near-random integration feature of viral cell engineering is risky and can cause integration at harmful sites, necessitating elaborate and expensive quality control procedures. Additionally, hiPSCs are difficult to transduce, with transgenes prone to epigenetic silencing-mediated loss of expression over time, similar to progenitor cell transduction.^25^ Gene editing represents a favorable alternative to viral transduction.^26^ Reporter transgenes are directly integrated into hiPSCs at a known location within the genome, ensuring consistent reporter expression in all daughter hiPSCs and differentiated progeny, without requiring cell transduction. The adeno-associated virus integration site 1 (*AAVS1*) locus situated within intron 1 of the protein phosphatase 1 regulatory subunit 12C (*PPP1R12C*) gene is considered a desirable locus for transgene incorporation due to flanking insulator elements shielding transgenes from inactivation or transactivation,^27^ making it particularly suitable for use in hiPSC modification. While transgene expression may impact *PPP1R12C* expression, adverse effects due to *AAVS1* disruption have not been reported^28^^,^^29^ and thus it is considered a safe harbor locus. In fact, several groups have reported successful transgene incorporation into *AAVS1* in hiPSCs, with expression retained after both long-term culture and differentiation to cells of multiple germ layers.^29^^,^^30^^,^^31^^,^^32^

Here, we used transcription activator-like effector nuclease (TALEN) gene editing to incorporate the hNIS-mGFP dual-modality reporter gene into the *AAVS1* safe harbor locus of a cGMP-compliant hiPSC line. We verified that AAVS1-hNIS-mGFP hiPSCs retain their pluripotency potential and capacity to differentiate into functional HLCs *in vitro* while retaining expression and function of the hNIS-mGFP reporter. Subsequently, we differentiated our AAVS1-hNIS-mGFP hiPSCs using an established protocol to produce multilineage liver buds (LBs), given that this candidate cell therapy approach has previously demonstrated capacity for long-term survival *in vivo*.^33^ We transplanted the reporter expressing LBs into the kidney capsules of healthy and liver injured mice and tracked their survival *in vivo* by repeat PET imaging. Imaging results were confirmed by histology and analysis of mouse sera for presence of human albumin.

## Results

### Gene editing and quality control of hNIS-mGFP-expressing hiPSCs

To deliver hNIS-mGFP into the *AAVS1* locus we exploited TALEN-mediated recombination. We generated a transgene construct with homology arms for *AAVS1*, AAVS1-CAG-hNIS-mGFP (see materials and methods, Figure S1), and confirmed its identity by Sanger sequencing. To establish hNIS-mGFP expressing hiPSCs, the CGT10 hiPSC line was passaged onto vitronectin, with additional pipetting-mediated mechanical disruption to achieve small colonies suitable for transfection.^10^^,^^23^^,^^34^ hiPSC colonies were co-transfected 24 h later with the AAVS1-CAG-hNIS-mGFP donor plasmid and the hAAVS1-TALEN left and right homology arm plasmid pair. After 48 h, live cell fluorescence microscopy identified distinct hNIS-mGFP expressing hiPSCs (Figure S2A). Crucially, hiPSC colonies seemed to maintain their pluripotent state as tightly compacted colonies with well-defined borders. Puromycin selection (Puro^R^ driven from *PPP1R12C* promoter) (Figure S2B) was used for initial selection followed by fluorescence-activated cell sorting for hNIS-mGFP-positive cells. After expansion of polyclonal hNIS-mGFP^+^ hiPSCs, we extracted genomic DNA for PCR screening and Sanger sequencing to confirm correct transgene insertion (Figure S2C). The TALEN-editing constructs generated for this study (Figure S1) used starting material from a previously published construct which has been successfully used to edit both hiPSCs and hESCs at the *AAVS1* to incorporate a reporter transgene,^35^ thus demonstrating that this editing strategy can be independently reproduced via different laboratories interested in a range of reporter approaches. To demonstrate the reproducibility of this approach to deliver the whole hNIS-mGFP cassette into the *AAVS1* locus for constitutive reporter expression in a hepatic context, we applied a similar process to that described for generating our CGT10.hNIS-mGFP transgenic hiPSC line to additionally edit HepG2 cells. HepG2 is an immortalized human liver cancer cell line that is commonly used as a control for pre-clinical studies of hepatic cell therapies due to its differentiated hepatic characteristics. HepG2-AAVS1-CAG-hNIS-mGFP cells were generated by co-transfection of the TALEN plasmids (Figure S3A) and antibiotic selection in the same manner as CGT10-hNIS-mGFP hiPSCs and characterization of polyclonal hNIS-mGFP^+^ HepG2s yielded similar results (Figure S3).

Next, we analyzed our CGT10.hNIS-mGFP hiPSC line for potential off-target mutations caused by TALEN mediated gene editing. Hockemeyer et al*.*^31^ have comprehensively analyzed sites with sequence similarity to the AAVS1-TALEN targeting site and reported that this AAVS1-TALEN pair has limited off-target mutations. The off-target site (OTS) identified as OTS10 was shown to have a higher incidence of mutation, and screening of this site has been used in other studies to assess the integrity of hiPSCs and hESCs genetically modified at the *AAVS1* locus.^35^ We opted for the same assessment with PCR amplification of OTS10 performed for both the CGT10.hNIS-mGFP line and unedited CGT10 hiPSCs. DNA fragments compared by electrophoresis showed the same expected size of OTS10, 167 bp (Figure S2D). Sanger sequencing of gel-purified PCR products showed no mutation in the transgenic hiPSCs relative to parental, unedited cells, indicating whether off-target mutations of functional significance were present in the CGT10.hNIS-mGFP line as a result of gene editing these were likely to be rare, given none were detected at the site with the highest likely incidence by Sanger sequencing (Figure S2E). Analysis of parental HepG2 cells at OTS10 relative to transgenic AAVS1-CAG-hNIS-mGFP HepG2s showed comparable results to hiPSCs (Figures S3D and S3E).

To detect incidences of rare off-target mutations, a higher resolution analysis of OTS10, along with two additional off-target sites OTS3 and OTS16 with sequence similarity to the TALEN pair (selected based on the original SELEX screen^31^) was performed via targeted deep sequencing. Genomic DNA of CGT10.hNIS-mGFP and unedited CGT10 hiPSCs were amplified via PCR with primers for the off-target regions (Table S2). DNA fragments compared by electrophoresis showed the same expected sizes for both parental and edited hiPSCs (OTS3, 221 bp; OTS10, 232 bp; OTS16, 201 bp; including Illumina adaptor sequences) (Figure S4). Next-generation sequencing was performed on DNA from gel extracted bands. Trimmed, quality control-filtered reads from parental and edited cells were aligned to the expected human genome reference sequence for the respective off-target regions. Results demonstrated a small number of rare indels (totaling <1%–5% of aligned reads) (Table S4). In the case of OTS10 these rare mutants were not previously detected by Sanger sequencing. While these results are higher than reported by Hockemeyer et al.^31^ for the same binding sites, they incorporate sequencing analysis of a larger region both upstream and downstream of the TALEN binding sites than previously assessed. Notably, indel rates were comparable between parental and edited cells for each site relative to the human reference sequence suggesting that a limited proportion of indels detected in CGT10.hNIS-mGFP hiPSCs relate to non-homologous end-joining. Introduction of mutagenic damage during PCR/DNA extraction protocols when preparing samples for indel/variant screening is also known to account for the majority of the erroneous identification of variants with low to moderate (1–5%) frequency.^36^ Comparable results for the same OTSs between parental HepG2s and AAVS1-CAG-hNIS-mGFP HepG2s were also seen (Figure S4 and Table S4). Overall, this high-resolution OTS analysis highlights the suitability of this approach for reporter gene integration during pre-clinical cell therapy development, but emphasizes the need for substantial deep sequencing where gene editing approaches are used in development of hiPSC lines suitable for clinical off-the-shelf clinical use.

### CGT10.hNIS-mGFP hiPSCs retain pluripotency potential and hepatic maturation capacity

While we previously demonstrated that lentivirus-mediated hNIS-mGFP expression did not impair the hepatic phenotype in HLCs,^23^ it was necessary here to assess whether CGT10.hNIS-mGFP hiPSCs retained the hepatic differentiation potential of unedited, parental CGT10 hiPSCs. Therefore, we explored three avenues to assess HLC differentiation potential: (1) expression of pluripotency markers in hiPSCs, (2) analysis of intermediate hepatic progenitor cell mRNA expression, and (3) phenotypic and functional analysis of mature HLCs.

**Figure 1:**
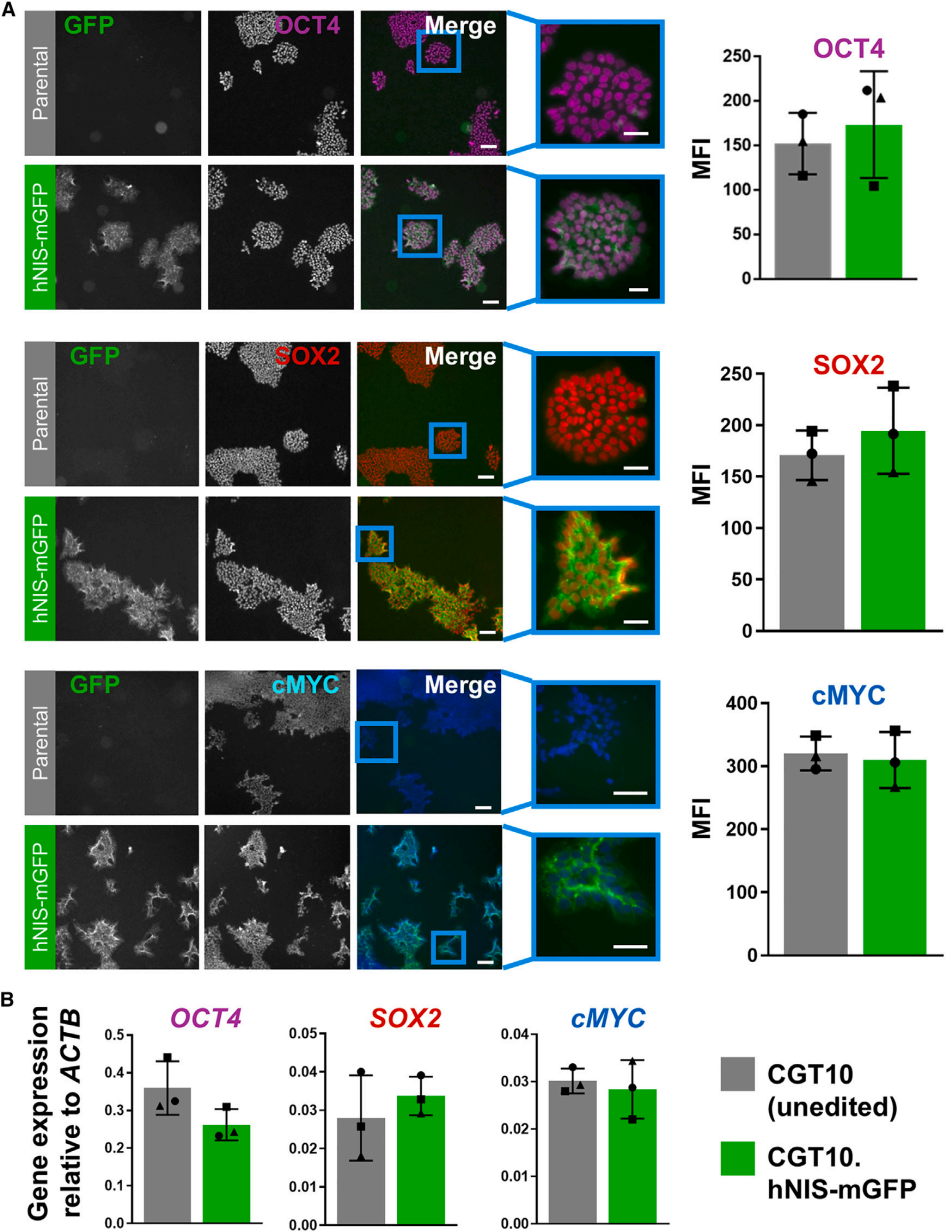
hNIS-mGFP reporter integration into the AAVS1 locus of CGT10 hiPSC did not impair pluripotency marker expression (A) CGT10.hNIS-mGFP or CGT10 colonies were seeded onto vitronectin-coated black 96-well plates, maintained for 3–5 days, fixed and immunostained for OCT4, SOX2, or cMYC before imaging. Representative fluorescence micrographs from n = 3 independent immunostaining experiments are shown; scale bars, 100 μm. Immunostaining was quantified by MFI (see materials and methods) and was performed on n > 50,000 cells per well (with triplicate wells per independent experiment); data points in quantification panels to the right represent independent experiments. (B) Total RNA was extracted from the same hiPSC batches as in (A) and *OCT4*, *SOX2*, and *cMYC* mRNA expression quantified by qPCR (ΔCt method with sample threshold cycle [Ct] values normalized to the housekeeping gene β-actin [ACTB]); n = 3 independent experiments (quadruplicate technical replicates per experiment). Statistical analyses by two-tailed Student's *t-*test showed no difference in mean MFI or gene expression (*p* > 0.05) between CGT10.hNIS-mGFP and CGT10 cells. All error bars shown are SD.

To assess expression of pluripotency markers, parental and CGT10.hNIS-mGFP hiPSCs were immunostained for octamer-binding transcription factor 4 (OCT4), sex determining region Y-box 2 (SOX2), and c-Myc (MYC) (Figure 1A). Immunostaining micrographs were segmented to define nuclear regions and mean fluorescence intensities (MFI) of the nuclear region in each cell were determined and reported as average MFI for all nuclei above background for each well (Figure 1A). The MFI showed no overall difference in pluripotency marker expression compared with parental CGT10 cells. To verify immunostaining results, total RNA was extracted from hiPSCs across the same three passages and mRNA expression of these pluripotency markers analyzed by qPCR. Results support the findings from immunostaining, showing no significant difference in mRNA expression between gene-edited and parental hiPSCs (Figure 1B), suggesting that expression of the hNIS-mGFP transgene does not impact on pluripotency marker expression.

**Figure 2:**
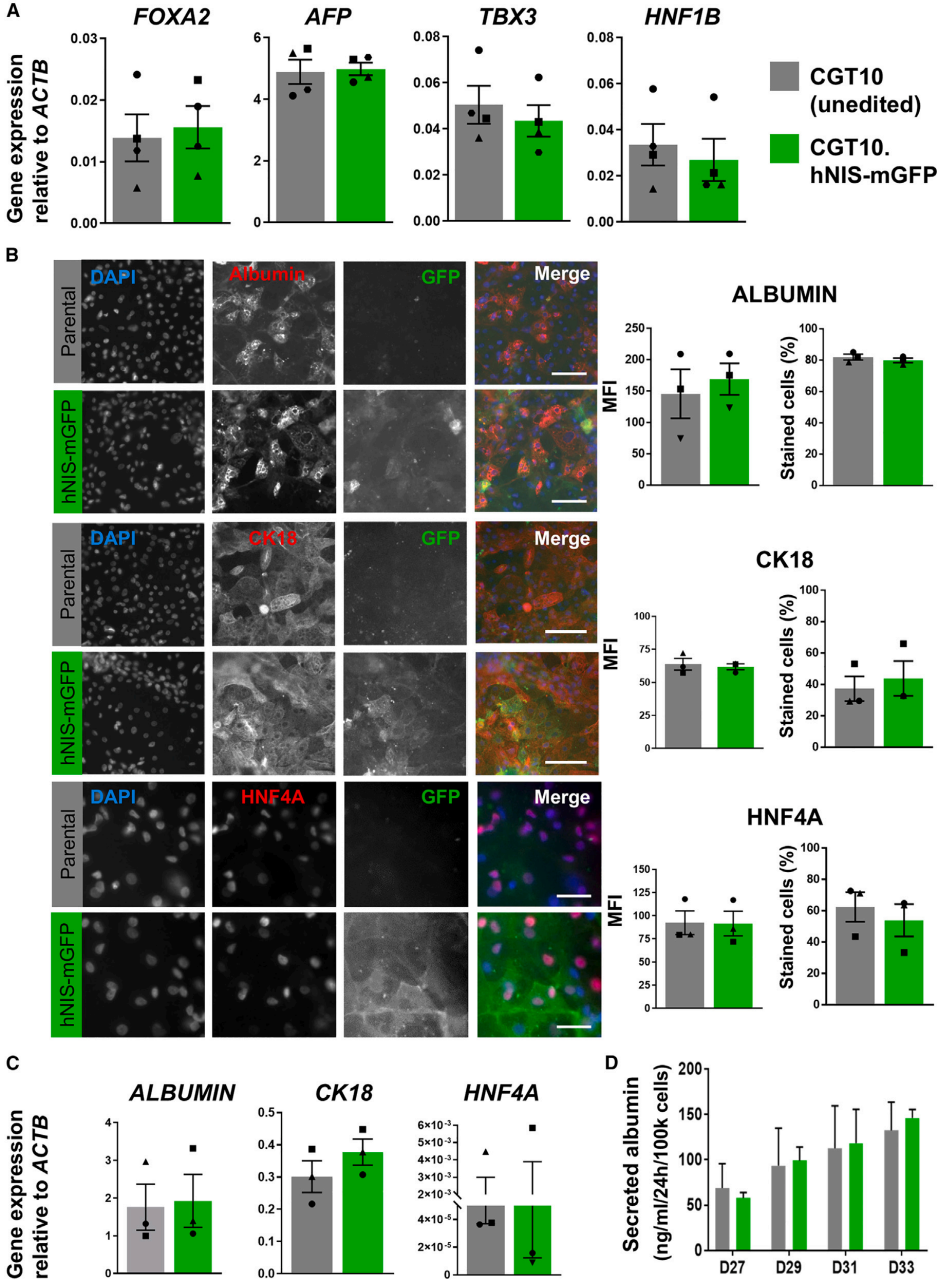
Integration of hNIS-mGFP into the AAVS1 locus results in CGT10 hiPSCs that retain differentiation capacity (A) Progenitor cells at day 13 of differentiation (D13) were collected, total RNA extracted and mRNA expression analyzed by qPCR for the hepatic progenitor genes Forkhead Box A2 (*FOXA2*), α-fetoprotein (*AFP*), T-box3 (*TBX3*), and HNF factor 1β (*HNF1B*); results are normalized to the housekeeping gene β-actin (*ACTB*; *cf.* Methods). Results are from n = 4 independent biological replicates (each with three technical repeats). Student's *t*-test indicated no difference in mean expression (*p* > 0.05; error bars ± SD). (B) D13 progenitors were passaged onto collagen-I coated plates for further maturation to D33 HLCs. HLCs were fixed, immunostained for mature hepatocyte markers, i.e., albumin, CK18, or HNF4A, and imaged. Representative fluorescence micrographs from n = 3 independent immunostaining experiments are shown; scale bars 100 μm. Immunostaining was quantified by MFI and proportion of positively stained cells (see materials and methods) and performed on n > 25,000 cells per well (with triplicate wells per independent experiment); error bars ± SEM. (C) HLC D33 qPCR analysis as in (A) but for markers of (B); n = 3 independent experiments (triplicate technical replicates per experiment); statistics as in (A). (D) Mean albumin secretion into culture media during HLC maturation analyzed by ELISA; n = 3 independent differentiations (triplicate technical replicates per experiment) results showed no difference at indicated differentiation time points (*p* > 0.05; multiple Student's *t-*test for each different time point and controlled for false discovery by two-stage step-up method of Benjamini, Krieger and Yekutieli); error bars are SD. Symbols indicate different differentiation batches (▴,▪,●,⬣).

To assess the impact of reporter expression on hepatic differentiation capacity, parental CGT10 and CGT10.hNIS-mGFP cells were differentiated in parallel and subjected to phenotypic and functional analyses. We assessed hepatic lineage specification of HLC progenitor cells by examining expression of four established surrogate genes known to be either critical to hepatic specification or expressed highly in fetal (rather than adult) hepatocytes; these were Forkhead Box A2, α-fetoprotein, T-box3, and hepatocyte nuclear factor (HNF) 1β. Results showed heterogeneity in expression of these hepatic progenitor marker genes between independent batches of differentiation, but with no overall difference observed between CGT10 parental and CGT10.hNIS-mGFP cells on day 13 after initiation of differentiation. This demonstrated comparative levels of differentiation proceeding to this point in both lines (Figure 2A).

Finally, the phenotypes of mature HLCs differentiated from either CGT10.hNIS-mGFP or unedited CGT10 were compared. We passaged hepatic progenitors onto collagen I at day 13 and matured them until day 33 when we analyzed their mature hepatocyte marker expression, first, by immunostaining for HNF4α, albumin, and cytokeratin-18 (CK18), and second, by qPCR analysis for the same markers. Resultant fluorescence micrographs (Figure 2B, left) showed cells from both lines had the expected polyhedral morphology of mature HLCs and expressed the markers as expected. Quantification (see materials and methods) revealed some variation in average expression between batches of differentiation (as seen in the progenitors), but no significant difference in MFI or percentage of positively stained cells between HLCs derived from unedited CGT10 and CGT10.hNIS-mGFP cells (Figure 2B, right). To verify immunostaining results, total RNA was extracted from cells collected from matched differentiations and marker expression analyzed by qPCR (Figure 2C). While variation in marker expression was also observed between independent differentiation batches in this analysis there was no difference in average expression between HLCs derived from parental and reporter expressing hiPSCs. Furthermore, we analyzed albumin secretion by differentiating HLCs, whereby we found a trend toward increased albumin secretion as maturation progressed, but importantly, without significant differences in the concentration of secreted albumin between parental and reporter expressing HLCs (Figure 2D). These data suggested that, while there were batch-to-batch variations in maturity, a known challenge in PSC-derived cell therapy translation, a high proportion of mature cells was consistently obtained with each differentiation irrespective of reporter expression. Comparable hepatic phenotypes were also demonstrated in transgenic AAVS1-hNIS-mGFP and parental HepG2s, supporting these conclusions (Figure S5).

### Reporter subunits are functional in CGT10.hNIS-mGFP hiPSCs and CGT10.hNIS-mGFP HLCs

**Figure 3:**
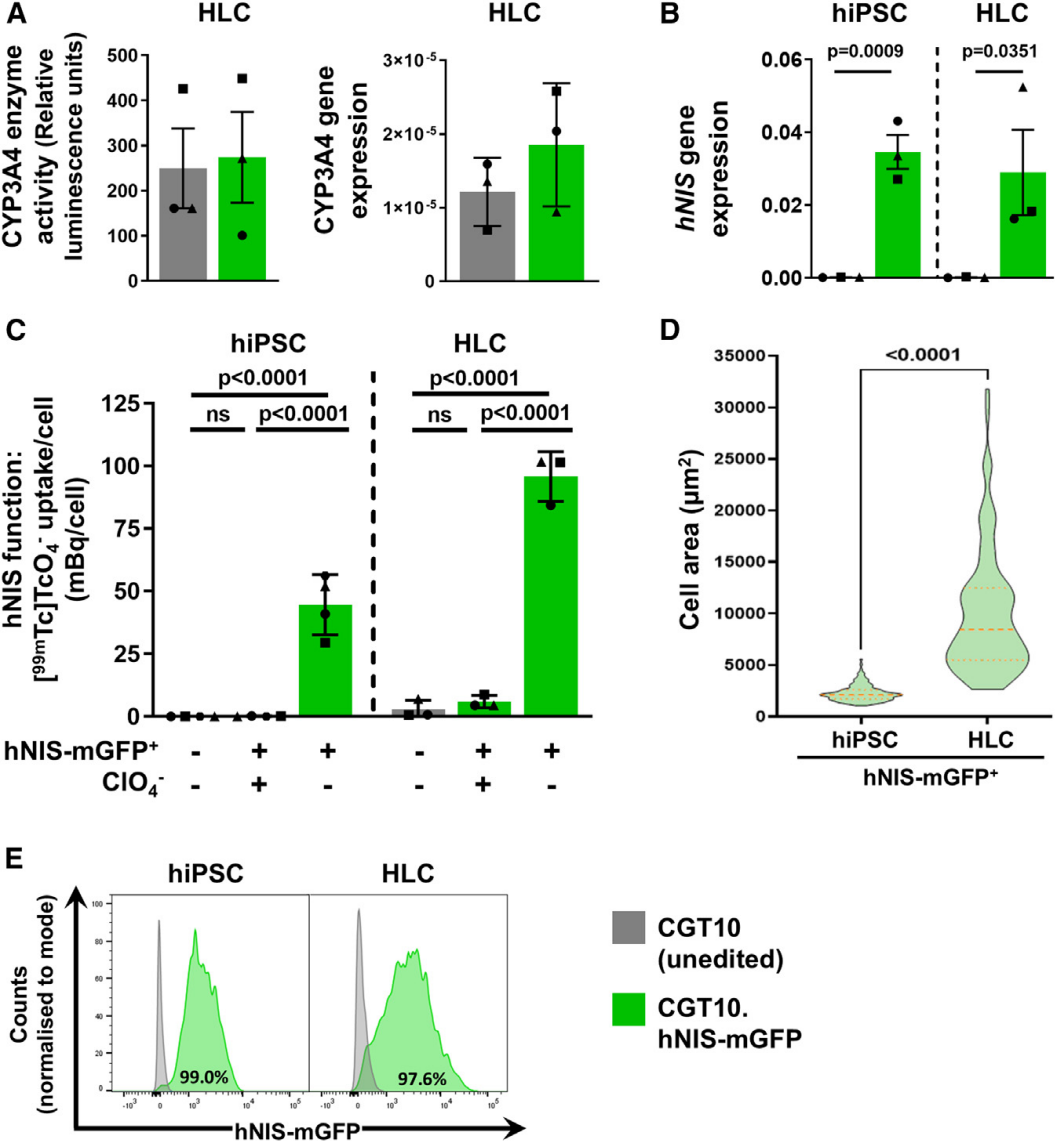
Verification of hNIS-mGFP expression and function in hiPSCs and functionally mature HLCs (A, left) CYP3A4 enzyme activity of mature HLCs (D33) and (A, right) CYP3A4 gene expression analysis by qPCR. Results show the average of *N* = 3 independent differentiations (triplicate technical replicates per differentiation); Student's *t-*test indicated no difference in mean expression (*p* > 0.05; error bars are SD) for both (A) and (B). (B) *hNIS* gene expression analysis by qPCR. Results show the average of n = 3 independent biological replicates (triplicate technical replicates each); error bars represent SD. The Student's *t*-test was used to determine statistical significance. (C) hNIS-mGFP function quantified by [^99m^Tc]TcO_4_^−^ uptake is shown in indicated cells either expressing hNIS-mGFP due to gene editing or in parental unedited cells. The hNIS co-substrate perchlorate (ClO_4_^−^) served as a [^99m^Tc]TcO_4_^−^ uptake specificity control in reporter expressing cells. n = 4 (for hiPSC) or n = 3 (for HLC) biological replicates (triplicate technical replicates for each biological sample); error bars SD and statistical significance determined by one-way ANOVA with Tukey’s multiple comparison correction. (D) Violin plot demonstrating the difference in cell size in hiPSCs versus HLCs in 2D *in vitro* culture, pooled data from n = 3 independent biological replicates (differentiations/passages) are shown (>100 cells per differentiation stage analyzed). Statistical significance determined by Mann-Whitney test. Orange dotted lines represent median and quartiles. (E) Representative flow cytometry histograms showing reporter expression distribution within a polyclonal population comparing CGT10 with CGT10.hNIS-mGFP hiPSC and subsequently differentiated HLCs with GFP^+^% indicated within histogram plot.

We confirmed the expression and function of the mGFP portion of our dual-mode reporter in both gene edited hiPSC and subsequently differentiated HLCs (Figures 1A and 2B). Function of the radionuclide reporter portion, hNIS, relies on correct plasma membrane insertion to enable substrate transport into cells. To validate this in mature hNIS-mGFP^+^ HLCs, we first verified hepatic functional maturity, and then performed radiotracer uptake assays with the hNIS substrate [^99m^Tc]TcO_4_^−^. Hepatic maturity as per cytochrome P450 3A4 (CYP3A4) enzyme activity showed variations in average CY3A4 activity between independent differentiation batches suggesting variation in the maturity of the HLCs, but with no significant differences in enzymatic activity between HLCs derived from parental CGT10 and CGT10.hNIS-mGFP cells, thereby demonstrating differentiation in both lines was comparable (Figure 3A, left). This was supported by CYP3A4 mRNA qPCR analysis of the same cells (Figure 3A). We also used qPCR to assess *hNIS* expression in the gene-edited hiPSC cells and mature HLCs (Figure 3B). [^99m^Tc]TcO_4_^−^ uptake assays showed that both hNIS-mGFP^+^ hiPSCs and differentiated hNIS-mGFP^+^ HLCs readily took up [^99m^Tc]TcO_4_^−^, and that this radiotracer uptake was sensitive to the competitive substrate perchlorate (Figure 3C), thereby demonstrating uptake to be hNIS specific. These results were again supported by analogous findings for hNIS expression and radiotracer uptake capacity in hNIS-mGFP HepG2s (Figures S6A and S6B). Greater radiotracer uptake per cell was observed in transgenic HLCs relative to transgenic hiPSCs (Figure 3C), and we attributed this to the larger size of HLCs compared hiPSCs (Figure 3D). The latter results, together with fluorescence microscopy data (Figures 1A and 2B) and representative flow cytometry data (Figure 3E), demonstrated that our hNIS-mGFP dual-mode reporter was fully functional in both reporter gene-engineered hiPSCs and mature HLCs (as well as in transgenic HepG2s) (Figures S5 and S6C).

### Generation of traceable multilineage LB organoids from CGT10.hNIS-mGFP hiPSC

Multilineage LB organoids are intended for use in clinical trials to treat congenital liver disorders, they can be manufactured at scale,^33^ and differentiated via GMP compatible protocols using chemically defined and animal origin-free media.^37^ This renders LB an ideal candidate approach to demonstrate the *in vivo* tracking potential of differentiated cell types derived from our CGT10.hNIS-mGFP hiPSC. We used the established protocol for generation of all-iPSC-derived LB organoids as previously reported,^33^ aiming to recapitulate the morphogenetic and signaling processes of early liver organogenesis to produce mature LB capable of vascularization. We independently differentiated three progenitor cell populations in 2D from hiPSCs, i.e., hepatic endodermal (HE) cells, septum transverse mesenchymal (STM) cells, and endothelial progenitor cells, each of which had adopted the expected morphology 10 days after the start of separate differentiation (Figure S7). Their identities were confirmed by qPCR and flow cytometry using appropriate vascular, mesenchymal, and hepatic specified endodermal markers (Figure S8) and subsequently the three donor-matched populations were combined and cells self-condensed overnight to form LB *in vitro* (Figure S9A). LB exhibited an initially tightly packed structure following 24 h of co-culture, with less sharply defined edges becoming evident over time as well as a progressive increase in the average area covered by individual LB (Figure S9B), demonstrating continued proliferation. Expression of hNIS-mGFP was maintained throughout LB expansion and maturation (Figure S9A) and analysis of LB mRNA expression *in vitro* up to day 18 of co-culture suggested increasing hepatic maturity relative to HE (Figure S9C), which was supported functionally by their increasing human albumin secretion into culture media (Figure S9D). Additionally, analysis of mesenchymal and endothelial markers after *in vitro* culture confirmed retained presence of these supporting cell populations (Figures S9E and S9F). The presence of hepatic and endothelial cell populations within LBs was also demonstrated by immunostaining of LB for hepatic (albumin, CK18) and endothelial markers (VE-cadherin, CD31) (Figure S10). Together, LB characterization results were in line with expectations based on previous hiPSC lines capable of consistent functional hepatic differentiation in 2D and being capable of forming functional LBs.^33^^,^^38^

### *In vivo* imaging of CGT10.hNIS-mGFP-derived multi-lineage LB in healthy mice

**Figure 4:**
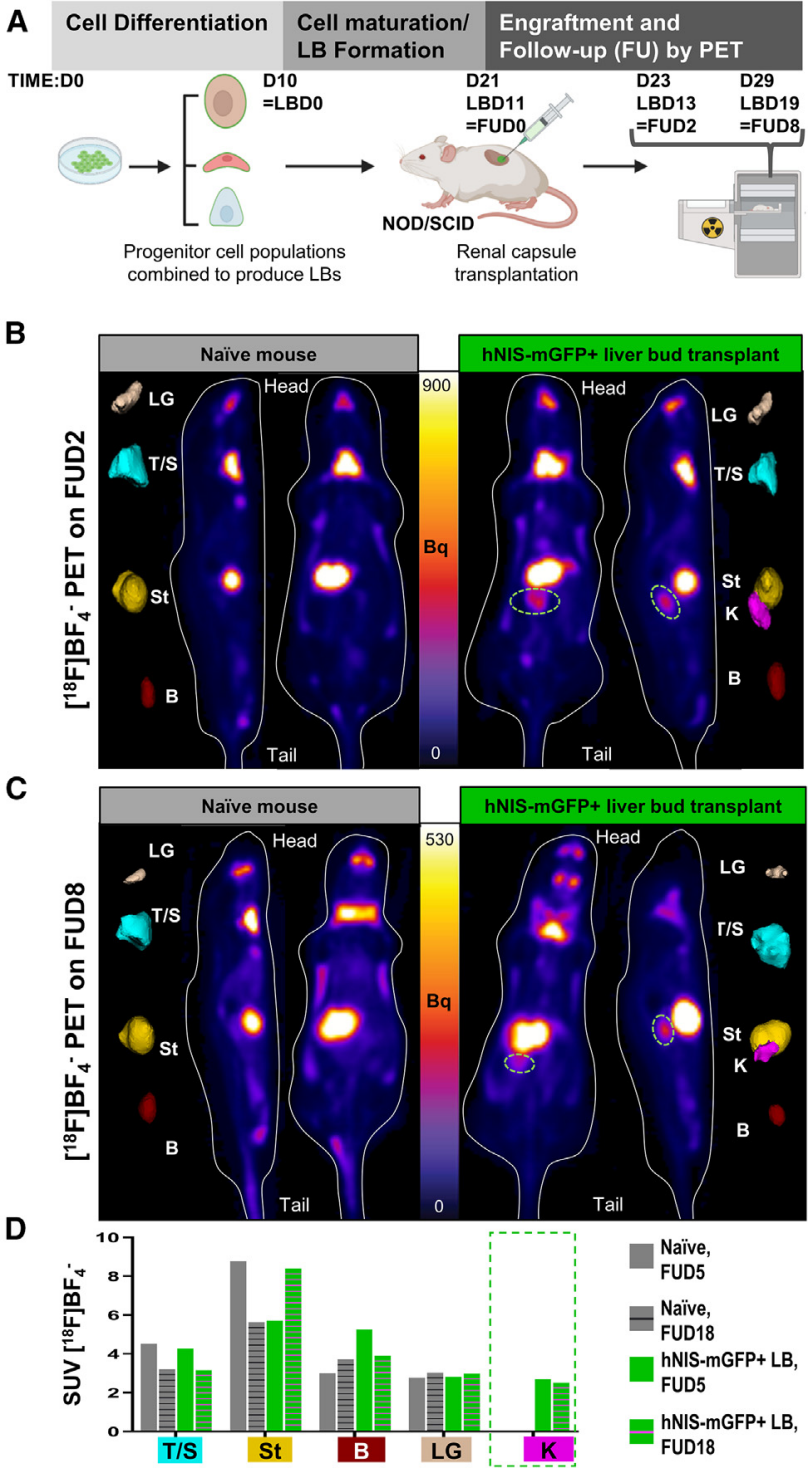
Repeat PET imaging of subrenally transplanted hNIS-mGFP^+^ LBs in a healthy mouse (A) Experimental scheme of the pilot experiment to determine LB *in vivo* detection by PET in a healthy NOD-SCID mouse. CGT10.hNIS-mGFP+ cells were differentiated into three lineages as monocultures, then mixed *in vitro* and condensed to form LBs (see materials and methods and Figure S7). LBs were transplanted into the subrenal capsule and subsequently, animals were *in vivo* imaged using the PET radiotracer 18FBF_4_^−^ to detect NIS-expressing LBs and tissues as indicated. (B and C) PET imaging of naive control and hNIS-mGFP^+^ LB-transplanted mice at FUD2 (B) and FUD8 (C) after LB transplantation. Sagittal and coronal sections are shown. In pseudocolors to the left and right, respectively, relevant organs with high radiotracer uptake are shown as 3D volumetric renderings after Otsu-afforded thresholding to enable segmentation. Segmented regions correspond to regions of endogenous mouse NIS expression and uptake including lacrimal glands (LG; sepia), thyroid and salivary glands (T/S; cyan), the stomach (St; yellow) as well as the bladder (B; red) representing not endogenous NIS expression but the renal radiotracer excretion route. In the hNIS-mGFP^+^ LB-transplanted animals, a clear signal in the kidney region onto which the LBs were transplanted is visible (green circle; K; magenta) at both timepoints demonstrating feasibility of imaging/tracking as well as the presence of viable hNIS-mGFP^+^ LB. (D) SUV quantified from *in vivo* PET images in (B and C) for the indicated organs after segmentation.

We chose the subrenal capsule as an established anatomical cell transplant niche suitable for evaluating feasibility of LB *in vivo* imaging by PET because it offers a pocket within which transplanted cell therapies are spatially confined, allowing for straightforward verification of surviving grafts and subsequent *ex vivo* histological evaluation complementing *in vivo* imaging data. CGT10.hNIS-mGFP-derived LB were subrenally transplanted into an immunocompromised non-obese diabetic mouse (NOD-SCID), as they were previously reported to facilitate high levels of human cell engraftment without rejection.^39^ Two days after LB transplantation (follow-up day 2 [FUD2]), 18FBF_4_^−^-afforded NIS-PET imaging was performed, with a naive NOD SCID mouse used as an imaging control (Figure 4). PET imaging demonstrated expected radiotracer uptake in organs not relevant to LB transplantation as a result of known endogenous murine NIS expression in these organs/tissues, including in the lacrimal glands, salivary glands, thyroid, and stomach.^40^ Radiotracer excretion also resulted in bladder signals; however, we could also unequivocally detect signal in the kidney after radiotracer administration demonstrating LBs were both detectable and viable, which was confirmed through the absence of signals in kidneys in the naive animal. Re-imaging the same animals on FUD8 after transplantation (FUD8) (Figure 4C) demonstrated continued survival of the LB. Image quantification between imaging sessions (via standardized uptake value [SUV]) revealed comparable radiotracer uptake in the kidney containing LB (SUV[FUD2] = 2.69 vs. SUV[FUD8] = 2.52). To validate that the hepatic cells in the LB were functional, we analyzed mouse serum collected on FUD 8 and found 10.6 ng/mL human albumin, supporting the PET imaging finding that LB remained viable. These data demonstrated feasibility to *in vivo* PET track CGT10.hNIS-mGFP-derived multi-lineage LB and showed their survival in this model.

### The potential to track CGT10-hNIS-mGFP derived LBs by PET imaging in liver-injured mice

**Figure 5:**
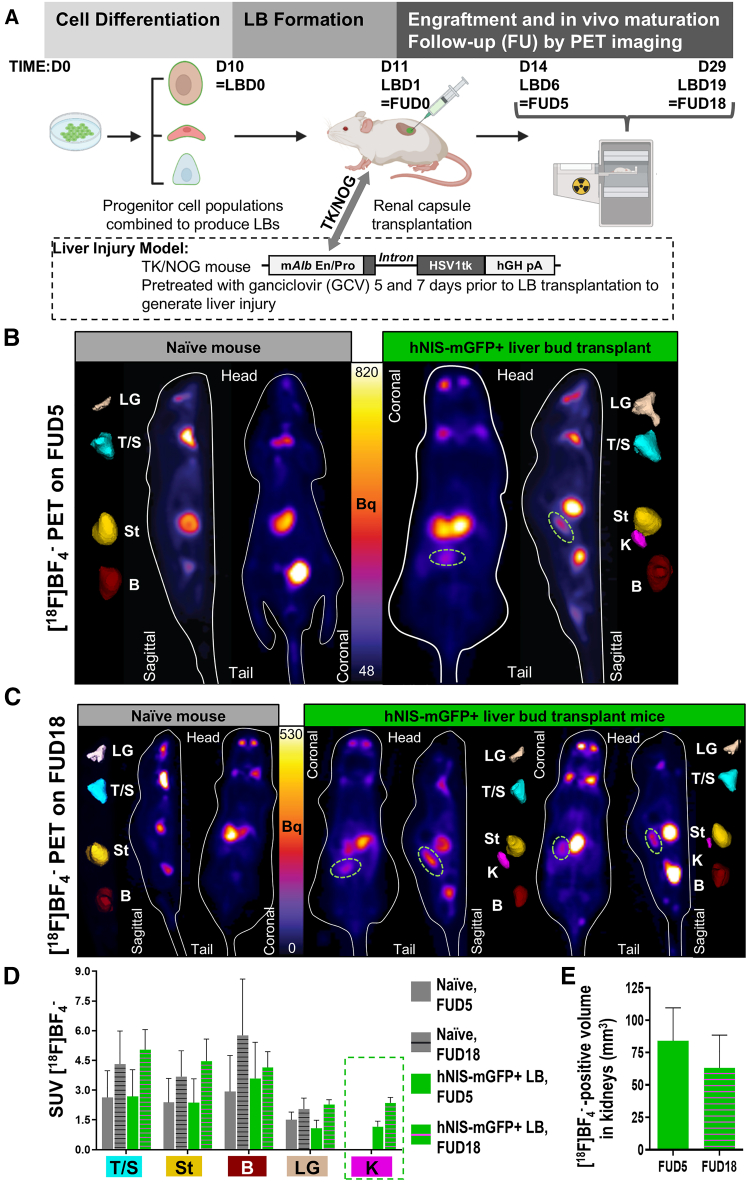
Repeat PET imaging of subrenally transplanted hNIS-mGFP+ LBs in TK-NOG mice (A) Experimental scheme of experiments to determine repeat LB *in vivo* detection by PET in liver injured TK-NOG mice. CGT10.hNIS-mGFP+ cells were differentiated into three lineages as monocultures, then mixed *in vitro* and condensed to form LBs (see materials and methods and Figure S7). TK-NOG mice were pre-treated with GCV 5 and 7 days before LB transplant to induce liver injury. Subsequently, LBs were transplanted into subrenal capsules and animals were PET imaged *in vivo* as indicated. (B and C) PET imaging of naive control and hNIS-mGFP+ LB-transplanted mice at FUD5 (B) and FUD18 (C) after LB transplantation. Representative sagittal and coronal sections are shown. Pseudocolors are relevant organs with high radiotracer uptake shown as 3D volumetric renderings after Otsu-afforded thresholding enabled segmentation. Segmented regions correspond to endogenous mouse NIS expression and uptake including lacrimal glands (LG; sepia), thyroid and salivary glands (T/S; cyan), the stomach (St; yellow) as well as the bladder (B; red) representing not endogenous NIS expression but the renal radiotracer excretion route. In the hNIS-mGFP^+^ LB transplanted animals, a clear signal in the kidney region where LBs were transplanted is visible (green circle; K; magenta) at both timepoints demonstrating feasibility of imaging/tracking and the presence of viable hNIS-mGFP^+^ LBs. (D) SUVs quantified from *in vivo* PET images in (C) for the indicated organs after segmentation, n = 2 mice per group, error bars represent SEM. (E) OTSU thresholded volumetric quantification of *in vivo* PET signals from kidneys of animals transplanted with traceable LBs error bars represent SEM.

**Figure 6:**
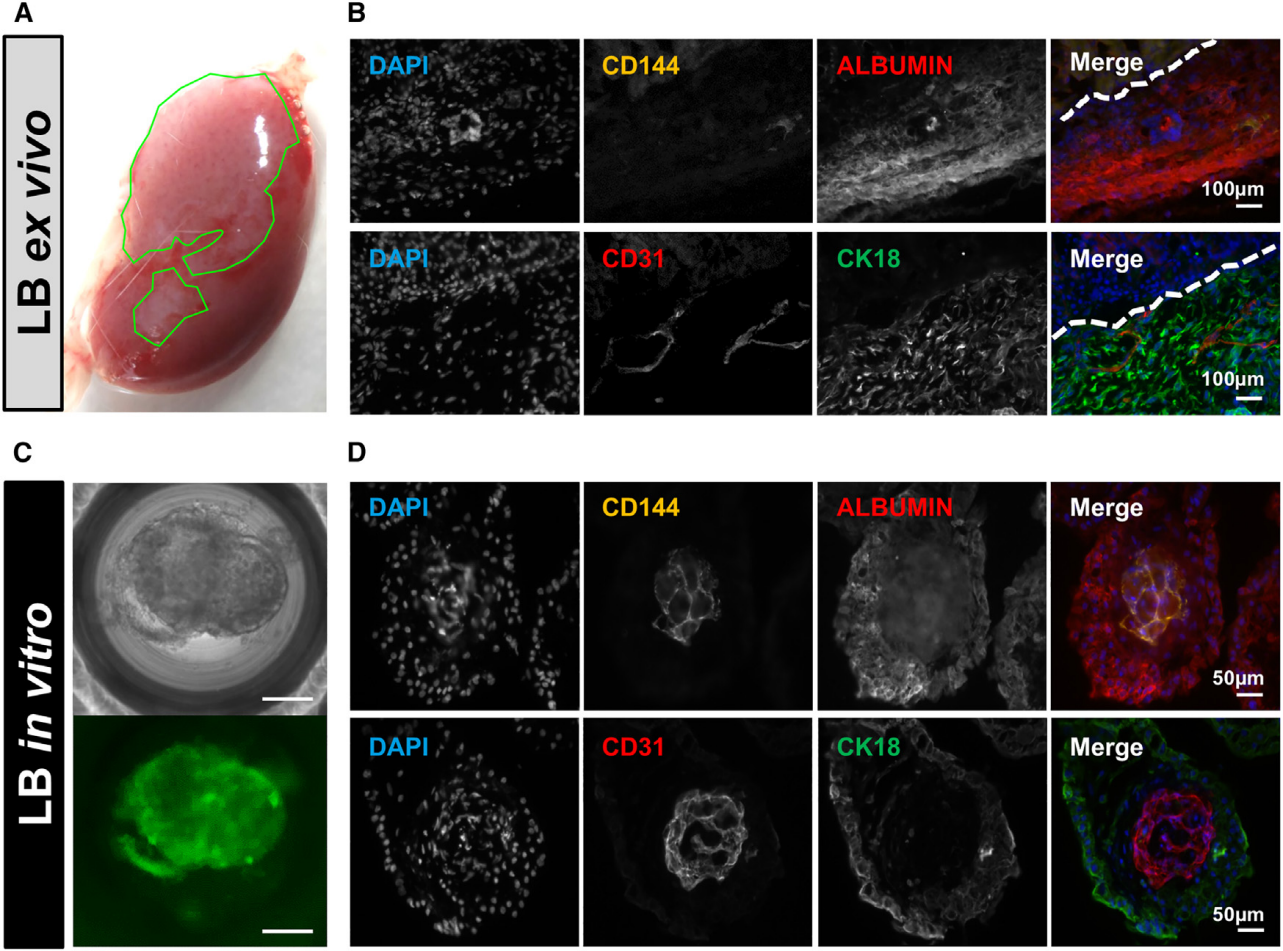
Comparative immunostaining of *ex vivo* harvested and *in vitro* cultured LB (A) Example of harvested kidney (18 days after hNIS-GFP+ LB transplantation; *cf.*Figure 5). The green encircled areas are visibly different due to LB transplantation. (B) Histology and immunostaining with antibodies raised against indicated antigens (anti-human: CD144, albumin, CD31, and CK18) and a nuclear stain to visualize cells (DAPI). Pseudocolors in the merged image use colors indicated in individual image descriptors. The border of the LB-transplanted area is indicated with a dashed line. Top and bottom rows represent adjacent sections from the same harvested kidney as in (A). (C) Brightfield and fluorescence widefield microscopy (GFP channel) of living hNIS-GFP^+^ LB in its culture dish. (D) Immunostaining of LB as in (B); rows represent adjacent sections from the same fixed LB population.

Next, we aimed to demonstrate the utility of the CGT10-hNIS-mGFP line for determining the *in vivo* survival of hiPSC-derived LB in a pre-clinical liver injury model. Therefore, we used subrenal capsule transplantation of LB into the TK-NOG mouse, which expresses the herpes simplex virus 1 thymidine kinase (HSV1-*tk*) driven by the mouse albumin enhancer/promoter (mAlb En/Pro; Figure 5A) within the liver of the highly immunodeficient NOG mouse.^41^ Administration of ganciclovir (GCV) to TK-NOG mice therefore enables targeted ablation of their HSV1-*tk*-expressing hepatocytes resulting in liver injury. After murine injury induction, we transplanted freshly prepared LBs (1 day after co-culture, rather than following an extended *in vitro* maturation period (as in Figure 4A), to ensure engraftment at the point when LB retain maximal proliferative potential, thereby enabling *in vivo* LB maturation (Figure 5A). To support comparisons in serum albumin concentration between experiments in Figures 4 and 5, the same differentiation endpoint was kept as a terminal imaging time point and experimental endpoint. PET imaging showed signals in the LB transplanted kidney (SUV = 1.2 ± 0.2; radioactive signal volume rendering: 84.2 ± 27.2 mm^3^) on FUD5 (Figure 5B), which were absent in the naive control TK-NOG (which had also been subjected to GCV-induced liver injury). On FUD18, we re-imaged the animals (Figure 5C), with results clearly indicating transplanted LB survival up to this time point with radioactivity signals within kidneys detected (SUV = 2.4 ± 0.3) (Figure 5D) also permitting volume rendering (63.2 ± 25.3 mm^3^) (Figure 5E). Detected human albumin in the serum of mice at sacrifice ranged from zero (naive mice) to clearly detectable amounts in LB transplanted TK-NOG mice (10.8 ± 0.1 ng/mL and 37.9 ± 0.9 ng/mL), with the results being comparable with those measured in the NOD-SCID LB transplanted mouse without liver injury (Figure 4). Upon harvesting, a prominent graft was visible in the subrenal capsule (Figure 6A), and tissue was subsequently fixed for immunostaining. LB from the same transplantation batch were maintained *in vitro* throughout the duration of the *in vivo* experiment and immunostained alongside LB-transplanted kidney sections (harvested on FUD18). Both *ex vivo* (Figure 6B) and *in vitro* (Figures 6C and 6D) LB showed comparable staining of human endothelial markers (VE-cadherin/CD144 and CD31) and human mature hepatic markers (albumin and CK18). Cells positively stained for human VE-cadherin and human CD31, both *in vitro* and *in vivo*, and demonstrated a morphology representative of a vascular network within the surrounding hepatic cell populations. The batch of *in vitro* cultured LB was additionally subjected to qPCR analysis for the same markers supporting immunostaining results (Figure S9). Together, the *in vivo* imaging data, the *ex vivo* serum-based albumin determinations, and the corresponding histology alongside the immunostaining and qPCR data from *in vitro* cultured LB from the same batch, demonstrate that hNIS-GFP^+^ LB retained their differentiated status throughout the whole *in vivo* experiment including *in vivo* maturation within the toxic microenvironment of this liver injury model. Moreover, our data reveal that providing sufficient initial LB engraftment and survival differentiated hepatic function is retained.

## Discussion

Primary HTxs have demonstrated promise for clinical use as an alternative or bridging therapy for OLT; however, wider implementation has been hampered by availability of high-quality primary hepatocytes and therapeutic transience. PSC-derived liver cell therapies offer the potential to provide an unlimited alternative source of high-quality cells. The high cost of developing PSC therapies acts as the main factor hindering their clinical translation.^42^ Methods for assessing the safe and effective delivery of the cells to the disease/injury site, ensuring effective cell engraftment and/or function at the injury site and preventing immunogenic host responses, which may reduce efficacy or result in graft transience are also increasingly vital to address translational bottlenecks. If administered cells can be non-invasively monitored over time, many of the pre-clinical development bottlenecks can be tackled. *In vivo* imaging can reduce animal cohort sizes and costs by monitoring the same animals over time, which is significant for pre-clinical therapy development. Combining imaging with repeat serum sample collection and complementary endpoint histological data will enhance the power of safety studies. Longitudinal imaging will also allow outstanding challenges, such as identifying the window in which graft loss/failure might occur or determining whether novel cell formats (e.g., organoids^33^ or hepatocyte sheets^43^) are more effective than more traditional single-cell approaches to be addressed. For clinical translation and *in vivo* monitoring of administered cell products, one means to tackle these challenges is the integration of a clinically compatible imaging reporter to facilitate longitudinal, non-invasive, and quantitative whole body *in vivo* imaging of transplanted cells.

Here, we have demonstrated that the incorporation of a radionuclide reporter gene within the AAVS1 safe harbor locus of a cGMP compliant hiPSC line (Figures S1 and S2) using TALEN gene editing. To facilitate pre-clinical research, we used our previously established dual-mode radionuclide-fluorescence hNIS-mGFP reporter, however, omission of the mGFP moiety would render the approach clinically compatible, too. Notably, the corresponding NIS radiotracers, e.g., [^99m^Tc]TcO_4_^−^ for SPECT imaging (radioisotope half-life [t_H_] = 6.0 h) or 18FBF_4_^−^ for PET imaging (t_H_ = 1.8 h)^44^^,^^45^^,^^46^ have already been clinically translated and are either in routine clinical use or readily accessible at PET centers, respectively. Few previous studies have incorporated radionuclide reporter cassettes into hPSCs using a gene editing approach to enable non-invasive cell tracking *in vivo*.^19^^,^^47^ In studies where this approach has been adopted, *in vivo* radionuclide imaging was restricted to monitoring transplanted PSC teratoma growth without transplanting and monitoring *in vitro* differentiated PSC-derived cell populations.^19^ Moreover, these authors used the radiotracer iodide-124, limiting repeat imaging opportunities (due to the long t_H_ of 4.26 days) and causing long intervals between repeat-imaging sessions (normally at least 5× t_H_ are needed); the latter is necessary to allow previously administered radiotracer to decay and thereby enable sensitive repeat-imaging, which is a prerequisite for cell therapy tracking.

Our CGT10.hNIS-mGFP hiPSC line enabled pre-clinical tracking of its differentiated progeny. To our knowledge, this study is the first to modify a cGMP-compatible hiPSC line at the AAVS1 locus to incorporate the hNIS-mGFP fusion reporter gene cassette for pre-clinical research, demonstrate differentiation and function of these transgenic cells toward the hepatic lineage following reporter integration, as well as image them longitudinally by PET. Our work here adds an additional dimension to the use of hNIS (alone or as a hNIS-fluorescent protein fusion reporter for pre-clinical research) in that it validates its suitability for hiPSCs and a differentiated downstream multi-lineage cell therapy model. Our *in vitro* characterization results showed that the reporter does not impact hiPSC pluripotency potential (Figure 1). Additionally, maturation proceeded as expected through an extended hepatic differentiation protocol (Figure 2). These data are in line with our previous findings, where mature CGT10 HLCs expressing lentivirally transduced hNIS-mGFP showed no difference in albumin or HNF4α expression relative to untransduced controls.^23^ Both parts of the reporter fusion also retained functionality after differentiation (Figure 3), which was particularly important for the radionuclide reporter portion (hNIS) that requires correct integration into the plasma membrane for function.^48^ Importantly, the radiotracer uptake per cell of CGT10.hNIS-mGFP derived HLCs was highly consistent across independent differentiations (with a range of only 17.4 mBq between independent uptake assays and mean ± SEM of 95.86 ± 5.75 mBq), this is beneficial compared with our previous work using lentiviral transduction mediated hNIS-mGFP expression where, although uptake was higher (due to multiple copy numbers per cell), it was also much more variable (as each differentiated batch of cells was transduced at the immature HLC stage of differentiation). The uptake range per HLC due to lentiviral transduction mediated hNIS-mGFP expression spanned 520.2 mBq across independent uptake assays/transduction batches, with a mean ± SEM of 1,085 ± 161.2 mBq.^23^ The gene editing approach therefore offers the substantial advantage of facilitating comparisons between *in vivo* experiments (e.g., comparing quantification of average engraftment) in a more straightforward and replicable manner. Given that significant variability in initial engraftment of HTx remains one of the major hurdles for wider utilization of this therapeutic approach, consistent tracer uptake per cell to enable quantitative, real-time high-resolution *in vivo* tracking is a valuable step forward for this methodology. Overall, our findings in the CGT10.hNIS-mGFP hiPSC line are supported by others using *AAVS1* gene edited transgenic PSCs, who have demonstrated retention of their reporter gene (e.g., fluorescent proteins such as GFP) across multiple passages as well as following differentiation to mature functional cell types, suggesting that incorporation of transgenes at this locus is generally compatible with retained pluripotency and differentiation capacity.^19^^,^^20^^,^^29^^,^^30^^,^^31^^,^^32^^,^^35^^,^^47^^,^^49^

To highlight the versatility and potential benefits of this reporter gene integration approach we used the gene-edited hiPSC line to enable a candidate liver cell therapy model (LB)^33^^,^^37^ to be tracked non-invasively *in vivo.* In our previous study, wherein hNIS-mGFP^+^ HLCs were intrahepatically transplanted, we were able to detect viable hNIS-mGFP^+^ cells after 24 h, but not at later timepoints due to lack of engraftment.^23^ Consequently, we adopted here a different model, namely transplantation of hNIS-mGFP^+^ LB into the kidney capsules of either healthy NOD-SCID mice (to demonstrate *in vivo* detectability by PET imaging) (Figure 4) or liver injured TK-NOG mice (Figure 5). In both cases, we were able to demonstrate the *in vivo* tracking potential by repeat PET imaging, notably up to 18 days after transplantation of LBs in the liver injury model. hNIS-mGFP^+^ LB transplanted kidneys of NOD-SCID and TK-NOG mice showed a consistently higher SUV (SUV of 1.2–6) than naive control mice and same-animal control (not transplanted) kidneys (SUV of 0). Engrafted LBs demonstrated retention of their differentiated cell phenotype after transplantation, evident by the presence of human albumin in mouse sera and as determined by the relevant markers via immunostaining (Figure 6).

The presented data on the CGT10.hNIS-mGFP cell line are proof of principle for gene editing and downstream characterization of hiPSCs when rendering them *in vivo* traceable by expression of an imaging reporter from a safe-harbor locus. We also provided an application example of hiPSC differentiation in the context of liver cell therapies, further demonstrating multi-lineage differentiation capabilities to be retained without notable impact of the engineered imaging reporter on expected phenotypes. As off-the-shelf PSC therapies approach more mainstream (pre-)clinical realisations,^50^^,^^51^^,^^52^ our approach and, more generally the need for use of constitutive imaging reporters, are evident requirements in the toolkit required to overcome bottlenecks in therapy development and translation to the clinics. Our work specifically unlocks the longitudinal assessments of biodistribution, engraftment, and long-term survival of a variety of hiPSC-derived cell types, which is of significant interest to the wider cell therapy and regenerative medicine field because of its ability to help overcome safety concerns and support therapy monitoring needs during clinical translation.

## Materials and Methods

### Reagents

Unless otherwise stated, reagents, standard chemicals, and plasticware was from either Millipore, Thermo Fisher Scientific, VWR, Nunc, Sarstedt, or TPP. [^99m^Tc]TcO_4_^−^ was generator eluted and supplied by Guy’s and St. Thomas’ Hospital Radiopharmacy (King’s College) and used within two half-lives. 18FBF_4_^−^ was supplied by the Department of Radiology, Yokohama City University Hospital and was synthetized by the fluorine exchange method as described previously^16^ including radiotracer quality control by high-performance liquid chromatography.

### Cell culture

Human embryonic kidney 293T cells (HEK293T; ATCC) were cultured in DMEM with 1 g/L of glucose supplemented with 10% (v/v) fetal bovine serum, penicillin (100 IU/mL) and streptomycin (0.1 mg/mL), and 2 mM *L*-glutamine. Cells were incubated at 37°C in a humidified atmosphere containing 5% (v/v) CO_2_.

Human iPSC colonies from the cGMP-derived CGT-RCiB-10 (working cell bank) hiPSC line (CGT10; from Cell and Gene Therapy Catapult) as well as its modified line, CGT-RCiB-10-AAVS1-CAG-hNIS-mGFP as generated in this work (CGT10-hNIS-mGFP) were maintained and differentiated as previously described.^10^^,^^23^^,^^34^

### Generation of constructs necessary for TALEN-mediated gene editing

Building on the AAVS1-CAG>hrGFP plasmid (Addgene #52344, gift from Dr Barcellos-Machado^35^), we replaced hrGFP with hNIS-mGFP to generate the AAVS1-CAG>hNIS-mGFP plasmid (see Figure S1). Therefore, hNIS-mGFP was amplified by PCR with flanking *HpaI* and *ClaI* restriction sites from the template plasmid pLNT SFFV>hNIS-mGFP(A206K)^15^; PCR primers were:

HpaI_hNIS forward: AAAGTTAACCACCATGGAGGCCGTGGAGACCG and

mGFP_ClaI reverse: CCCCCCATCGATTTACTTGTACAGCTCGTCC.

Generated DNA was subcloned into pCR-Blunt (ZeroBlunt PCR cloning kit; Thermo Fisher Scientific), bacterially amplified, digested with *HpaI* and *ClaI*), and subcloned into the AAVS1-CAG>hrGFP backbone after hrGFP removal (using *HincII* and *ClaI*). Clones were bacterially amplified, screened (*via* digest with *EcoRI*), and correct clones confirmed by Sanger DNA sequencing.

### Transient transfection of AAVS1 CAG>hNIS-mGFP to verify reporter gene expression

We seeded 2 × 10^5^ HEK293T cells/well in a 12-well plate 24 h before transfection and washed twice with PBS. We mixed 1 μg AAVS1 CAG>hNIS-mGFP plasmid DNA with 3 μg linear 25 kD polyethyleneimine in 50 μL serum-free DMEM, incubated for 15 min at room temperature (RT), and the mix then added dropwise to HEK293T cells in 0.5 mL complete DMEM. At 48 h after transfection, live cell fluorescence microscopy (EVOS AMEFC4300 equipped with filter cubes appropriate for GFP) revealed transgene expression.

### TALEN gene editing of hiPSCs

A previously published hiPSC lipofectamine transfection protocol was adapted for compatibility with our feeder-free culture platform.^53^ CGT10 hiPSC colonies were seeded at low density as small colonies onto vitronectin-coated 12-well plates and incubated for 24 h. We used 0.4 μg hAAVS1 TALEN-Left and 0.4 μg hAAVS1 TALEN-Right plasmids (Addgene #52341 and #52342, respectively, gift from Dr Barcellos-Machado) together with 1 μg AAVS1-CAG>hNIS-mGFP donor plasmid (in 50 μL OptiMEM with 2 μL Lipofectamine Stem Transfection Reagent (Thermo Fisher Scientific), incubated at RT for 15 min and supplemented with E8 medium (Gibco) for transfecting CGT10 cells per well. We added 200 μL E8 medium to each well and CGT10 cells were kept under normal culture conditions for 5 h and then 500 μL E8 medium was added. The medium was exchanged 24 h later for fresh E8 medium. Duplicate transfections were performed, and un-transfected wells were kept as growth/toxicity controls. Live cell fluorescence microscopy was used 48 h after transfection to identify GFP expression within colonies (Leica DM IL inverted microscope with Leica DFC3000G camera and filter cube appropriate for GFP). Transgene-positive cells were selected with puromycin-supplemented E8 medium; wherein medium was replaced daily with increasing puromycin concentration (0.5–1.0 μg/mL) over 5 days of culture.

### Analysis of gene-edited cells

Genomic DNA (gDNA) from parental CGT10 hiPSCs or CGT10-hNIS-mGFP hiPSCs was isolated (Qiagen QIAmp DNA mini kit) according to manufacturer’s instructions. To confirm correct transgene insertion into the *PPP1R12C* locus, we used analytic PCR with primers targeting either the *AAVS1* locus and a portion of the transgene, or regions within hNIS (Table S1). For preliminary analysis of potential nucleotide insertion/deletion events or transgene integration at genomic locations outside of the *AAVS1* target locus (i.e., OTS), PCR was performed using primers targeting the top-ranked OTS based on SELEX-derived base frequency matrices, as described previously (OTS10, OTS10-forward, and OTS10-reverse primers).^31^^,^^35^ Subsequently, three of the top-ranked OTSs (OTS3, OTS10, and OTS16) were subjected to targeted deep-sequencing to gain a higher resolution understanding of any potential rare indel events. PCR reactions were performed with gDNA to amplify the OTSs of interest with Illumina partial adapters incorporated 5’ (Table S2). For PCR, Q5 High-Fidelity DNA polymerase (NEB) was used in a final volume of 25 μL with primers at final concentrations of 0.5 μM each and 100 ng of template gDNA per reaction, unless otherwise indicated. The thermocycling protocol used is detailed in Table S3. PCR products supplemented with Gel Loading Dye were separated on 0.8% agarose gels in TAE buffer, using SYBR Safe DNA Gel stain (1 μL dye/10 mL gel). DNA from bands of the expected sizes (i.e., approximately 1 kb for hNIS transgene expression analyses and approximately 167 bp for initial OTS10 OTS analyses) were isolated from gels using the Wizard SV Gel and PCR Clean-Up System (Promega) according to manufacturer’s instructions. To confirm nucleotide sequences for correct transgene insertion and preliminary OTS analysis, extracted DNA was sent for Sanger Sequencing (GENEWIZ) using the same primers as PCR reactions. For targeted deep sequencing, extracted DNA of the expected sizes (OTS3, 221 bp; OTS10, 232 bp; OTS16, 201 bp) underwent next generation amplicon sequencing in 2 × 250 bp configuration (15,000–63,000 reads per sample, Illumina MiSeq) contracted out to GENEWIZ/Azenta (Amplicon-EZ service).

### Phenotyping of 2D-cultured HLCs and hiPSCs by immunofluorescence

For HLC analysis, day 13 immature cells were seeded onto collagen-I pre-coated black μClear 96-well plates (Greiner) at a density of 7 × 10^4^ cells/well to ensure a confluent monolayer formed. HLCs were subsequently cultured/matured under standard conditions until day 33 of the differentiation. For hiPSC staining, μClear 96-well plates were pre-coated with vitronectin and hiPSCs passaged as small colonies and maintained under standard culture conditions for 3–5 days (dependent on colony size).

Cells were washed once in PBS then fixed in 4% PFA for 15 min at RT and then washed three times with PBS for 5 min. For nuclear staining, cells were permeabilized by 0.5% Triton X-100 (v/v) in PBS for 10 min, washed thrice with PBS (5 min each) and blocked for 20 min at RT with PBS containing 1% BSA, 3% donkey serum, and 0.1% Triton X-100 prior. Cells were incubation with indicated primary antibodies for 1 h at RT. For HLC staining, goat anti-human albumin (A80-129A, Bethyl Laboratories, 10 μg/mL), rabbit anti-human HNF4α (clone EPR3648/ab92378, Abcam, 1.48 μg/mL), mouse anti-human CK18 (clone DC10/ab7797, Abcam, 10 μg/mL) was used. For hiPSC staining, mouse anti-human OCT-3/4 (Sc-5279 (C-10), Santa Cruz Biotechnology, 2 μg/mL), goat anti-SOX2 (AF2018, R&D Systems, 2 μg/mL), and rabbit anti-C-Myc (Sc-764 (*N*-262), Santa Cruz Biotechnology, 2 μg/mL) was used. Cells were then washed thrice in PBS (5 min each) and incubated with appropriate secondary antibodies for 40 min at RT; secondary antibodies were fluorophore-conjugated and raised against goat (AlexaFluor-647-conjugated), mouse (Alexa Fluor 647), rabbit (Alexa Fluor 647), and rabbit (Alexa Fluor 568) (all from Thermo Fisher Scientific and used 3 μg/mL; host for all was donkey). Cells were again washed thrice with PBS (5vmin each) and their nuclei stained with NucBlue (Life Technologies; dilution 1:25 in PBS). Images were acquired using a PerkinElmer Operetta or PerkinElmer Operetta CLS automated high content microscope using 10, 20, or 40× objectives contingent on cell size and with fluorescence filters appropriate for the dyes used.

To determine the MFI or the proportion of cells expressing a protein of interest in acquired fluorescence micrographs, Harmony software (v4.1 or 4.8, PerkinElmer) was used to set up appropriate image analysis pipelines. Representative pipelines for analysis of nuclear (hiPSC staining using antibodies targeting OCT4) and cytoplasmic staining (HLC staining of albumin) are depicted in Figures S12 and S13. The same analysis pipelines and parameters were used for all technical and biological replicates quantifying staining of the indicated antigen. Analysis was of triplicate/quadruplicate technical replicate wells per biological replicate, with results reported for triplicate independent biological replicates.

### Generation of multilineage LBs

Three distinct progenitor cell populations were differentiated from the CGT10>hNIS-mGFP hiPSC line in parallel for 10 days in 2D using the previously reported protocol^33^; these were HE, endothelial cell progenitors, and STM cells. LBs were produced by co-culturing these progenitor cells in micropore plates as described previously^33^ (see Figures S7 and S11 for graphical schemes).

### Assessment of LB area

To assess LB growth over time, the 2D area of collected LBs was measured using the IN-Cell Analyzer 2000 system (GE Healthcare). Overlapping fields of view from whole-well scanned brightfield images were stitched together using the IN-Cell Developer Toolbox software (GE Healthcare). LB areas were determined using the particle analysis function of Fiji ImageJ software (version 1.51n). Briefly, recorded 16-bit images were converted to binary masks, processed to remove debris and objects crossing the image borders (by adjusting size thresholds), and subjected to the 'watershed' function to delineate neighboring LBs. Subsequently, LB area was assessed using analyze particles (Analyze>Analyze particles). Reported area data for each well of LB was pooled in Prism Software v7 (GraphPad Inc.) to produce a frequency distribution graph for different timepoints.

### Fixation and embedding of *in vitro* maintained LB

LBs maintained *in vitro* were fixed at a matched time point to sub-renally transplanted LBs. Fixation and embedding aimed to maintain organoid structures for staining by minimizing mechanical disruption through adaptation of a previously detailed method.^54^

Briefly, LB were transferred from culture plates into 1.5-mL tubes by manual pipetting and allowed to sink by gravity. Culture media was aspirated. LB were washed with 1mL PBS and gravity-pelleted before PBS aspiration. Subsequently, LB were fixed in 4% PFA for 15 min and washed thrice in PBS (10 min/wash). LB were resuspended in 30% sucrose solution at 4°C overnight. The sucrose solution was aspirated and LB were suspended in 300 μL pre-warmed gelatin/sucrose solution (7.5%/10%, respectively, in PBS, 37°C). Tubes were incubated at 37°C for 15 min to allow equilibration and gravity pelleting. Gelatin/sucrose blocks were prepared by coating plastic embedding cassettes with gelatin/sucrose solution and incubating on wet ice to enable solidification. Equilibrated LB were pipetted as high-density droplets (approximately 20 μL each) onto the prepared blocks and incubated on wet ice until solidified. Warm gelatin solution was applied to the cassette until LB were submerged in a thin layer, then incubated further on wet ice until solidified. Blocks were frozen by suspension in liquid nitrogen vapor and stored at −80°C ahead of cryo-sectioning.

### Fixation and embedding of subrenally transplanted LB

Isolated kidneys were washed once in PBS and then immersed in 4% PFA for 16 h at 4°C. To maintain structural integrity during freezing, kidneys were dehydrated at 4°C by the following steps: 5% sucrose solution (1h), 15% sucrose solution (overnight), 30% sucrose solution (overnight). Kidneys were bisected (lateral border toward concave center of kidney) to maximize the number of sections across the capsule, and then embedded in optimal cutting temperature medium (OCT; VWR) within plastic embedding cassettes. Blocks were frozen by suspension in liquid nitrogen vapor and stored at −80°C.

### LB immunofluorescence microscopy

We cryo-sectioned 20-μm sections (*in vitro* cultured organoid gelatin blocks) or 7 μm sections (OCT kidney blocks) and adhered then onto MAS-coated slides (Matsunami). Embedding agents were removed from sections by either incubation of slides in PBS at 37°C for 20 min (gelatin blocks) or washing slides thrice in PBS for 5 min each (OCT blocks). LB were generously encircled with a hydrophobic delimiting pen. Blocking buffer (Protein Block Serum-Free ready-to-use, Dako) was applied to LB and sections incubated at RT for 2 h with slides subsequently washed three times with 0.05% Tween 20 in PBS (PBST). Staining included the following primary antibodies raised against the indicated antigens: human CD31 (Dako, M0823; host mouse); human CK8/18 (Progen, GP11; host guinea pig); human VE-Cadherin (R&D Systems, AF938; host goat); and human albumin (Sigma, A3293; host rabbit). Antibodies were prepared as 1:200 dilutions in PBST and sections incubated overnight at 4°C. Sections were washed thrice for 5min in PBST and incubated at RT for 40 min with the following fluorophore-conjugated secondary antibodies: goat (Alexa Fluor 555; host donkey), rabbit (Alexa Fluor 647; host donkey), guinea pig (Alexa Fluor 647; host goat), and mouse (Alexa Fluor 555; goat) (all from Thermo Fisher Scientific, staining concentration 3.75 μg/mL). After staining, sections were washed thrice for 5 min in PBST and mounted with coverslips using Apathy’s Mounting Media containing 1 μg/mL DAPI for nuclear staining. Samples were imaged using an Axio Imager.M1 microscope (Carl Zeiss) equipped with appropriate filter cubes to detect GFP, Alexa Fluor 555, and Alexa Fluor 647.

### Animals and surgical procedures

Generally, mice were maintained under sterile conditions according to the Yokohama City University institutional guidelines for the use of laboratory animals with sterile food and water available *ad libitum*. Mice were kept in individually ventilated plastic cages with environmental enrichment and bedding material. Cages were held in dedicated, licensed air-conditioned animal rooms, under light/dark cycles lasting 12 h. Maximum cage occupancy was five animals and fresh cages were supplied weekly.

Surgical procedures for subrenal transplantation were performed as previously described.^55^ For *in vivo* PET imaging experiments, animals were randomly assigned as naive imaging control (no transplant) or experimental animals, wherein LB were transplanted into the left subrenal capsules. The initial pilot to verify trackability was performed using 8- to 12-week-old female non-obese diabetic NOD/Shi-Scid Jic mice (NOD-SCID, Sankyo Lab, Tsukuba/Japan). For the liver injury model, 8-week-old male NOD.Cg-Prkdc^scid^ Il2rg^tm1Sug^Tg(Alb-UL23)7-2/ShiJic (TK-NOG,^41^ CLEA Japan) mice were administered 50 mg/kg GCV (dissolved in PBS) by intraperitoneal injection 7 days and 5 days before subrenal LB transplantation; control animals also received GCV. GCV is not ordinarily toxic to human or mouse tissues, but in the TK-NOG mouse the herpes simplex virus type 1 thymidine kinase (HSV1-*tk*) transgene is expressed under the mouse albumin enhancer/promoter (mAlb/En/Pro); therefore, GCV administration results in tissue-specific ablation of transgenic liver parenchymal cells. To confirm liver damage, blood serum levels of aspartate aminotransferase (AST) and alanine transaminase (ALT) were measured before LB transplantation using an automatic biochemical analyzer and DRI-CHEM 700V slides (FujiFilm Corporation, Japan) appropriate for AST and ALT analysis according to manufacturer’s instructions. Activity levels were confirmed to be within the expected range relative to mouse weight for the dosing regimen used (600–800 IU/L AST, 1100–1300 IU/L ALT).^41^ Humane endpoints for the study were indicated by weight loss, persistent hunched posture, and jaundice. Cohort sizes were selected to be sufficiently conclusive for proof-of-principle studies focused on the detectability of *in vivo* traceable cells, and with minimizing animal use in mind.

### Human albumin ELISA

The concentration of secreted human albumin in collected cell culture media or mouse sera was assessed using the Human Albumin Quantitation Set (Bethyl Laboratories) according to manufacturer’s instructions.

### RNA extraction and cDNA synthesis

RNA was extracted using the RNeasy MiniKit (Qiagen) according to manufacturer’s instructions. RNA concentration and purity of isolated RNA was determined with a NanoDrop spectrophotometer. Samples with a 260/280 ratio of ≥1.8, indicative of good quality RNA, were used for complementary DNA (cDNA) synthesis. cDNA was synthesized by reverse transcription using the SuperScript VILO cDNA Synthesis Kit (Invitrogen, hiPSCs and HLCs) or the High-Capacity cDNA Reverse Transcription Kit (Applied Biosystems, LBs and LB progenitors) according to manufacturer’s instructions.

### Real-time qPCR

Analysis of mRNA expression of hepatic and pluripotency genes was assessed by qPCR using the iTaq Universal SYBR Green Supermix (Bio-Rad) or the Thunderbird SYBR qPCR master mix (Toyobo). Cycle reactions were performed with a CFX384 Real-Time PCR System (Bio-Rad) or LightCycler 480 Instrument II Real-time PCR System (Roche) according to the conditions listed in Table S3. Gene-specific primers and probes used are detailed in Table S5. Technical replicates of sample threshold cycle (Ct) values were normalized to the human β-actin (ACTB) housekeeping gene or the eukaryotic 18S rRNA Endogenous Control reference (Applied Biosystems). Quantification for each independent experiment was by the 2^−ΔCt^ method, where ΔCt = meanC_t_^target gene^-meanC_t_^housekeeping gene^, thereby reporting the expression as relative to the internal control for all independent samples (allowing variation in expression between experiments to be reflected in results). Mean expression and SD relative to the housekeeping gene for triplicate biological replicates is detailed unless otherwise specified.

### *In vivo* imaging

Non-invasive PET imaging to detect NIS-expressing cells/tissues was performed on anesthetized mice (isoflurane, 1.5%–2.0% [v/v] in pure O_2_) using 5MBq 18FBF_4_^−^ (administered intravenously in 100 μL PBS) essentially according to protocols described previously.^56^ For animal PET scanning, an Inveon scanner (Siemens) equipped with Inveon Acquisition Workplace 1.5 Service Pack 1 and a 400–6,600 keVp window was used. Static PET scans were acquired for 30 min followed by 15-min transmission scans to enable attenuation correction. PET data were reconstructed using a 3D Ordered Subset Expectation Maximization (3DOSEM)-based iterative algorithm^57^ with corrections for attenuation, detector dead time, and radioisotope decay. Images were processed including quantitative analysis using VivoQuant software v3.5 (inviCRO). Volume rendering was performed using VivoQuant’s 3D implementation of Otsu’s thresholding method^58^ to separate signals from the background. The total activity in the whole animal (excluding the tail) at the time of radiotracer administration was defined as the injected dose. Radioactivity in each region of interest was quantified using VivoQuant and expressed as standardised uptake value (SUV).

### Statistics

Statistical analyses were performed using Prism software v7 (GraphPad). For the determination of statistical significance using *p* values, the significance level α was set to 0.05. Details are specified within text and figure legends.

## Data and code availability

The data presented here are available on request from the first or corresponding author. The animal imaging data are not publicly available due to a lack of repository and the size of the raw original datasets.

## Acknowledgments

The authors thank Professor Fiona M. Watt and the members of her lab past and present for their invaluable support in enabling elements of this work to be completed in the department formerly known as the Center for Stem Cells & Regenerative Medicine, King’s College London as well as Dr S. Tamir Rashid and the Cell and Gene Therapy Catapult UK for the use of the CGT10 parental line. The authors also thank the Isotope and Animal Research Centers at Yokohama City University, Dr. Takayoshi Oba, Professor Yun-Zhong Nie, and members of Professor Hideki Taniguchi laboratory for their support. Parts of the figures in this paper were produced using biorender.com.

The authors acknowledge funding from the following sources: Guy’s and St. Thomas’ Charity (PhD studentship to C.A.H.), the 10.13039/501100001691Japan Society for the Promotion of Science (Summer Program award to C.A.H.), the United Kingdom Regenerative Medicine Platform (C.A.H.), the University of Edinburgh College of Medicine & Veterinary Medicine Career Development Scheme (C.A.H.), and 10.13039/501100000289Cancer Research UK [C48390/A21153 to G.O.F.). This work was also supported by the Falk Transformational Awards Program, NIH Director’s New Innovator Award (DP2 DK128799-01) and AMED CREST (20gm1210012h0001), 10.13039/100000002NIH grant UG3/UH3 DK119982, PHS Grant P30 DK078392 (Integrative Morphology Core and Pluripotent Stem Cell and Organoid Core) of the Digestive Disease Research Core Center, 10.13039/100007449Takeda Science Foundation Award, 10.13039/501100004398Mitsubishi Foundation Award, and 10.13039/100009619AMED grants JP18fk0210037h0001, JP18bm0704025h0001, JP21gm1210012h0002, JP21bm0404045h0003, JP21fk0210060h0003, JST Moonshot JPMJMS2022-10 and JPMJMS2033-12, and 10.13039/501100001691JSPS KAKENHI Grant JP18H02800, 19K22416 (T.T.). This research was funded in part, by the 10.13039/100010269Wellcome Trust (Wellcome/EPSRC Center for Medical Engineering WT203148/Z/16/Z). For open access, the author has applied a Creative Commons Attribution (CC BY) license to any Author Accepted Manuscript version arising. This research was funded/supported by the 10.13039/501100000272National Institute for Health Research (NIHR) Biomedical Research Centre based at Guy’s and St Thomas' NHS Foundation Trust and King’s College London and/or the NIHR Clinical Research Facility. The views expressed are those of the author(s) and not necessarily those of the NHS, NIH, NIHR, or the Department of Health.

The study was conducted in accordance with the Declaration of Helsinki. The study involved animals, and all procedures related to animal work were performed in accordance with all legal, ethical, and institutional requirements (PPL70/7302) as dictated by UK legislation and committees at Yokohama City University. Sample sizes of *in vivo* experiments were based on prior work from our laboratories and kept to a minimum in line with law and ethical guidelines for animal research in the UK as were the *in vivo* endpoints.

## Author contributions

Conceptualization, C.A.H. and G.O.F.; methodology, C.A.H., H.A., T.T., and G.O.F.; validation, C.A.H., G.O.F., and T.A., YT (radiotracer synthesis); formal analysis, C.A.H., H.A., and G.O.F.; investigation, C.A.H., H.A., and E.Y.; resources, G.O.F., T.T., T.A., and Y.T.; data curation, C.A.H. and G.O.F.; writing—original draft preparation, C.A.H. and G.O.F.; writing—review and editing, all authors; visualization, C.A.H. and G.O.F.; supervision, G.O.F., H.A., and T.T.; project administration, C.A.H. and G.O.F.; funding acquisition, G.O.F., C.A.H., and T.T. All authors have read and agreed to the published version of the manuscript.

## Declaration of interests

T.T. is a scientific advisor to Healios KK. The funders had no role in the design of the study; in the collection, analyses, or interpretation of data; in the writing of the manuscript; or in the decision to publish the results.

## Supplemental information

Document S1. Figures S1–S13 and Tables S1–S5

Document S2. Article plus supplemental information

## References

[1] Williams R., Aspinall R., Bellis M., Camps-Walsh G., Cramp M., Dhawan A., Ferguson J., Forton D., Foster G., Gilmore I. (2014). Addressing liver disease in the UK: a blueprint for attaining excellence in health care and reducing premature mortality from lifestyle issues of excess consumption of alcohol, obesity, and viral hepatitis. Lancet.

[2] NHS Blood and Transplant (2019). Annual Report on Liver Transplantation: Report for 2018/2019 (1 April 2009 - 31 March 2019. https://nhsbtdbe.blob.core.windows.net/umbraco-assets-corp/16782/nhsbt-liver-transplantation-annual-report-2018-19.pdf.

[3] Branger P., Samuel U. (2018). Eurotransplant International Foundation Annual Report 2018.

[4] Kim W.R., Lake J.R., Smith J.M., Skeans M.A., Schladt D.P., Edwards E.B., Harper A.M., Wainright J.L., Snyder J.J., Israni A.K., Kasiske B.L. (2016). United States Organ Transplantation OPTN/SRTR Annual Data Report 2014: Liver. Am. J. Transplant..

[5] Rajvanshi P., Kerr a, Bhargava K.K., Burk R.D., Gupta S. (1996). Efficacy and safety of repeated hepatocyte transplantation for significant liver repopulation in rodents. Gastroenterology.

[6] Hansel M.C., Gramignoli R., Skvorak K.J., Dorko K., Marongiu F., Blake W., Davila J., Strom S.C. (2014). The history and use of human hepatocytes for the treatment of liver diseases: The first 100 patients. Curr. Protoc. Toxicol..

[7] Lee C.A., Sinha S., Fitzpatrick E., Dhawan A. (2018). Hepatocyte transplantation and advancements in alternative cell sources for liver-based regenerative medicine. J. Mol. Med..

[8] Kmieć Z. (2001).

[9] Ibars E.P., Cortes M., Tolosa L., Gómez-Lechón M.J., López S., Castell J.V., Mir J. (2016). Hepatocyte transplantation program: Lessons learned and future strategies. World J. Gastroenterol..

[10] Ashmore-Harris C., Fruhwirth G.O. (2022). Generation of In Vivo Traceable Hepatocyte-Like Cells from Human iPSCs. Methods Mol. Biol..

[11] Ashmore-Harris C., Iafrate M., Saleem A., Fruhwirth G.O. (2020). Non-invasive Reporter Gene Imaging of Cell Therapies, including T Cells and Stem Cells at Cell Press. Mol. Ther..

[12] Iafrate M., Fruhwirth G.O. (2020). How Non-invasive in vivo Cell Tracking Supports the Development and Translation of Cancer Immunotherapies. Front. Physiol..

[13] Volpe A., Lang C., Lim L., Man F., Kurtys E., Ashmore-Harris C., Johnson P., Skourti E., de Rosales R.T.M., Fruhwirth G.O. (2020). Spatiotemporal PET Imaging Reveals Differences in CAR-T Tumor Retention in Triple-Negative Breast Cancer Models. Mol. Ther..

[14] Jacob J., Nadkarni S., Volpe A., Peng Q., Tung S.L., Hannen R.F., Mohseni Y.R., Scotta C., Marelli-Berg F.M., Lechler R.I. (2021). Spatiotemporal in vivo tracking of polyclonal human regulatory T cells (Tregs) reveals a role for innate immune cells in Treg transplant recruitment. Mol. Ther. Methods Clin. Dev..

[15] Fruhwirth G.O., Diocou S., Blower P.J., Ng T., Mullen G.E.D. (2014). A Whole-Body Dual-Modality Radionuclide Optical Strategy for Preclinical Imaging of Metastasis and Heterogeneous Treatment Response in Different Microenvironments. J. Nucl. Med..

[16] Volpe A., Man F., Lim L., Khoshnevisan A., Blower J., Blower P.J., Fruhwirth G.O. (2018). Radionuclide-fluorescence Reporter Gene Imaging to Track Tumor Progression in Rodent Tumor Models. J. Vis. Exp..

[17] Maiques O., Fanshawe B., Crosas-Molist E., Rodriguez-Hernandez I., Volpe A., Cantelli G., Boehme L., Orgaz J.L., Mardakheh F.K., Sanz-Moreno V., Fruhwirth G.O. (2021). A preclinical pipeline to evaluate migrastatics as therapeutic agents in metastatic melanoma. Br. J. Cancer.

[18] Shi S., Zhang M., Guo R., Miao Y., Zhang M., Hu J., Xi Y., Li B. (2014). Feasibility of lentiviral-mediated sodium iodide symporter gene delivery for the efficient monitoring of bone marrow-derived mesenchymal stem cell transplantation and survival. Int. J. Mol. Med..

[19] Wolfs E., Holvoet B., Ordovas L., Breuls N., Helsen N., Schönberger M., Raitano S., Struys T., Vanbilloen B., Casteels C. (2017). Molecular Imaging of Human Embryonic Stem Cells Stably Expressing Human PET Reporter Genes After Zinc Finger Nuclease–Mediated Genome Editing. J. Nucl. Med..

[20] Ostrominski J.W., Yada R.C., Sato N., Klein M., Blinova K., Patel D., Valadez R., Palisoc M., Pittaluga S., Peng K.W. (2020). CRISPR/Cas9-mediated introduction of the sodium/iodide symporter gene enables noninvasive in vivo tracking of induced pluripotent stem cell-derived cardiomyocytes. Stem Cells Transl. Med..

[21] Punzón I., Mauduit D., Holvoet B., Thibaud J.L., de Fornel P., Deroose C.M., Blanchard-Gutton N., Vilquin J.T., Sampaolesi M., Barthélémy I., Blot S. (2020). In Vivo Myoblasts Tracking Using the Sodium Iodide Symporter Gene Expression in Dogs. Mol. Ther. Methods Clin. Dev..

[22] Templin C., Zweigerdt R., Schwanke K., Olmer R., Ghadri J.R., Emmert M.Y., Müller E., Küest S.M., Cohrs S., Schibli R. (2012). Transplantation and tracking of human-induced pluripotent stem cells in a pig model of myocardial infarction. Circulation.

[23] Ashmore-Harris C., Blackford S.J., Grimsdell B., Kurtys E., Glatz M.C., Rashid T.S., Fruhwirth G.O. (2019). Reporter gene-engineering of human induced pluripotent stem cells during differentiation renders in vivo traceable hepatocyte-like cells accessible. Stem Cell Res..

[24] Comisel R.M., Kara B., Fiesser F.H., Farid S.S. (2021). Lentiviral vector bioprocess economics for cell and gene therapy commercialization. Biochem. Eng. J..

[25] Müller-Kuller U., Ackermann M., Kolodziej S., Brendel C., Fritsch J., Lachmann N., Kunkel H., Lausen J., Schambach A., Moritz T., Grez M. (2015). A minimal ubiquitous chromatin opening element (UCOE) effectively prevents silencing of juxtaposed heterologous promoters by epigenetic remodeling in multipotent and pluripotent stem cells. Nucleic Acids Res..

[26] Ashmore-Harris C., Fruhwirth G.O. (2020). The clinical potential of gene editing as a tool to engineer cell-based therapeutics. Clin. Transl. Med..

[27] Ogata T., Kozuka T., Kanda T. (2003). Identification of an insulator in AAVS1, a preferred region for integration of adeno-associated virus DNA. J. Virol..

[28] Henckaerts E., Dutheil N., Zeltner N., Kattman S., Kohlbrenner E., Ward P., Clément N., Rebollo P., Kennedy M., Keller G.M., Linden R.M. (2009). Site-specific integration of adeno-associated virus involves partial duplication of the target locus. Proc. Natl. Acad. Sci. USA.

[29] DeKelver R.C., Choi V.M., Moehle E.A., Paschon D.E., Hockemeyer D., Meijsing S.H., Sancak Y., Cui X., Steine E.J., Miller J.C. (2010). Functional genomics, proteomics, and regulatory DNA analysis in isogenic settings using zinc finger nuclease-driven transgenesis into a safe harbor locus in the human genome. Genome Res..

[30] Oceguera-Yanez F., Kim S.I., Matsumoto T., Tan G.W., Xiang L., Hatani T., Kondo T., Ikeya M., Yoshida Y., Inoue H., Woltjen K. (2016). Engineering the AAVS1 locus for consistent and scalable transgene expression in human iPSCs and their differentiated derivatives. Methods.

[31] Hockemeyer D., Wang H., Kiani S., Lai C.S., Gao Q., Cassady J.P., Cost G.J., Zhang L., Santiago Y., Miller J.C. (2011). Genetic engineering of human pluripotent cells using TALE nucleases. Nat. Biotechnol..

[32] Hockemeyer D., Soldner F., Beard C., Gao Q., Mitalipova M., DeKelver R.C., Katibah G.E., Amora R., Boydston E.A., Zeitler B. (2009). Efficient targeting of expressed and silent genes in human ESCs and iPSCs using zinc-finger nucleases. Nat. Biotechnol..

[33] Takebe T., Sekine K., Kimura M., Yoshizawa E., Ayano S., Koido M., Funayama S., Nakanishi N., Hisai T., Kobayashi T. (2017). Massive and Reproducible Production of Liver Buds Entirely from Human Pluripotent Stem Cells. Cell Rep..

[34] Blackford S.J.I., Ng S.S., Segal J.M., King A.J.F., Austin A.L., Kent D., Moore J., Sheldon M., Ilic D., Dhawan A. (2019). Validation of a library of cGMP-compliant human pluripotent stem cell lines for use in liver therapy. Stem Cells Transl. Med..

[35] Qian K., Huang C.T.L., Chen H., Blackbourn L.W., Chen Y., Cao J., Yao L., Sauvey C., Du Z., Zhang S.C. (2014). A simple and efficient system for regulating gene expression in human pluripotent stem cells and derivatives. Stem Cell..

[36] Chen L., Liu P., Evans T.C., Ettwiller L.M. (2017). DNA damage is a pervasive cause of sequencing errors, directly confounding variant identification. Science.

[37] Sekine K., Ogawa S., Tsuzuki S., Kobayashi T., Ikeda K., Nakanishi N., Takeuchi K., Kanai E., Otake Y., Okamoto S. (2020). Generation of human induced pluripotent stem cell - derived liver buds with chemically defined and animal origin - free media. Sci. Rep..

[38] Takebe T., Zhang R.-R., Koike H., Kimura M., Yoshizawa E., Enomura M., Koike N., Sekine K., Taniguchi H. (2013). Vascularized and functional human liver from an iPSC-derived organ bud transplant. Nature.

[39] Shultz L.D., Schweitzer P.A., Christianson S.W., Gott B., Schweitzer I.B., Tennent B., McKenna S., Mobraaten L., Rajan T.V., Greiner D.L. (1995). Multiple defects in innate and adaptive immunologic function in NOD/LtSz-scid mice. J. Immunol..

[40] Cho J.-Y., Léveillé R., Kao R., Rousset B., Parlow A.F., Burak W.E., Mazzaferri E.L., Jhiang S.M. (2000). Hormonal Regulation of Radioiodide Uptake Activity and Na+/I- Symporter Expression in Mammary Glands. J. Clin. Endocrinol. Metab..

[41] Hasegawa M., Kawai K., Mitsui T., Taniguchi K., Monnai M., Wakui M., Ito M., Suematsu M., Peltz G., Nakamura M., Suemizu H. (2011). The reconstituted “humanized liver” in TK-NOG mice is mature and functional. Biochem. Biophys. Res. Commun..

[42] Neofytou E., O’Brien C.G., Couture L.A., Wu J.C. (2015). Hurdles to clinical translation of human induced pluripotent stem cells. J. Clin. Invest..

[43] Nagamoto Y., Takayama K., Ohashi K., Okamoto R., Sakurai F., Tachibana M., Kawabata K., Mizuguchi H. (2016). Transplantation of a human iPSC-derived hepatocyte sheet increases survival in mice with acute liver failure. J. Hepatol..

[44] O’Doherty J., Jauregui-Osoro M., Brothwood T., Szyszko T., Marsden P.K., O’Doherty M.J., Cook G.J.R., Blower P.J., Lewington V. (2017). 18F-tetrafluoroborate, a PET probe for imaging sodium/iodide symporter expression: Whole-body biodistribution, safety, and radiation dosimetry in thyroid cancer patients. J. Nucl. Med..

[45] Jiang H., DeGrado T.R. (2018). [^18^F]Tetrafluoroborate ([^18^F]TFB) and its analogs for PET imaging of the sodium/iodide symporter. Theranostics.

[46] Jiang H., Schmit N.R., Koenen A.R., Bansal A., Pandey M.K., Glynn R.B., Kemp B.J., Delaney K.L., Dispenzieri A., Bakkum-Gamez J.N. (2017). Safety, pharmacokinetics, metabolism and radiation dosimetry of 18F-tetrafluoroborate (18F-TFB) in healthy human subjects. EJNMMI Res..

[47] Wang Y., Zhang W.Y., Hu S., Lan F., Lee A.S., Huber B., Lisowski L., Liang P., Huang M., de Almeida P.E. (2012). Genome editing of human embryonic stem cells and induced pluripotent stem cells with zinc finger nucleases for cellular imaging. Circ. Res..

[48] Portulano C., Paroder-Belenitsky M., Carrasco N. (2014). The Na+/I- Symporter (NIS): Mechanism and medical impact. Endocr. Rev..

[49] Lopez-Yrigoyen M., Fidanza A., Cassetta L., Axton R.A., Taylor A.H., Meseguer-Ripolles J., Tsakiridis A., Wilson V., Hay D.C., Pollard J.W., Forrester L.M. (2018). A human iPSC line capable of differentiating into functional macrophages expressing ZsGreen: a tool for the study and *in vivo* tracking of therapeutic cells. Philos. Trans. R. Soc. Lond. B Biol. Sci..

[50] Deuse T., Hu X., Gravina A., Wang D., Tediashvili G., De C., Thayer W.O., Wahl A., Garcia J.V., Reichenspurner H. (2019). Hypoimmunogenic derivatives of induced pluripotent stem cells evade immune rejection in fully immunocompetent allogeneic recipients. Nat. Biotechnol..

[51] Lanza R., Russell D.W., Nagy A. (2019). Engineering universal cells that evade immune detection. Nat. Rev. Immunol..

[52] Crow D. (2019). Could iPSCs Enable “Off-the-Shelf” Cell Therapy?. Cell.

[53] Jacob A., Morley M., Hawkins F., McCauley K.B., Jean J.C., Heins H., Na C.L., Weaver T.E., Vedaie M., Hurley K. (2017). Differentiation of Human Pluripotent Stem Cells into Functional Lung Alveolar Epithelial Cells. Cell Stem Cell.

[54] Lancaster M.A., Knoblich J.A. (2014). Generation of cerebral organoids from human pluripotent stem cells. Nat. Protoc..

[55] Takebe T., Zhang R.-R., Koike H., Kimura M., Yoshizawa E., Enomura M., Koike N., Sekine K., Taniguchi H. (2014). Generation of a vascularized and functional human liver from an iPSC-derived organ bud transplant. Nat. Protoc..

[56] Diocou S., Volpe A., Jauregui-Osoro M., Boudjemeline M., Chuamsaamarkkee K., Man F., Blower P.J., Ng T., Mullen G.E.D., Fruhwirth G.O. (2017). [^18^F]tetrafluoroborate-PET/CT enables sensitive tumor and metastasis *in vivo* imaging in a sodium iodide symporter-expressing tumor model. Sci. Rep..

[57] Hudson H.M., Larkin R.S. (1994). Accelerated image reconstruction using ordered subsets of projection data. IEEE Trans. Med. Imag..

[58] Otsu N. (1979). A Threshold Selection Method from Gray-Level Histograms. IEEE Trans. Syst. Man Cybern..

