# Supplementary material for: Gene editing enables non-invasive in vivo PET imaging of human induced pluripotent stem cell-derived liver bud organoids
Source: Mol Ther Methods Clin Dev. 2025 Jan 7;33(1):101406. doi: 10.1016/j.omtm.2025.101406 (PMC11803834; doi:10.1016/j.omtm.2025.101406)
Supplement: Document S1. Figures S1–S13 and Tables S1–S5 [file mmc1.pdf]

**OMTM, Volume *33***

## Supplemental information

# Gene editing enables non-invasive *in vivo* PET imaging of human induced pluripotent stem cell-derived liver bud organoids

**Candice Ashmore-Harris, Hiroaki Ayabe, Emi Yoshizawa, Tetsu Arisawa, Yuuki Takada, Takanori Takebe, and Gilbert O. Fruhwirth**

## Figure S1

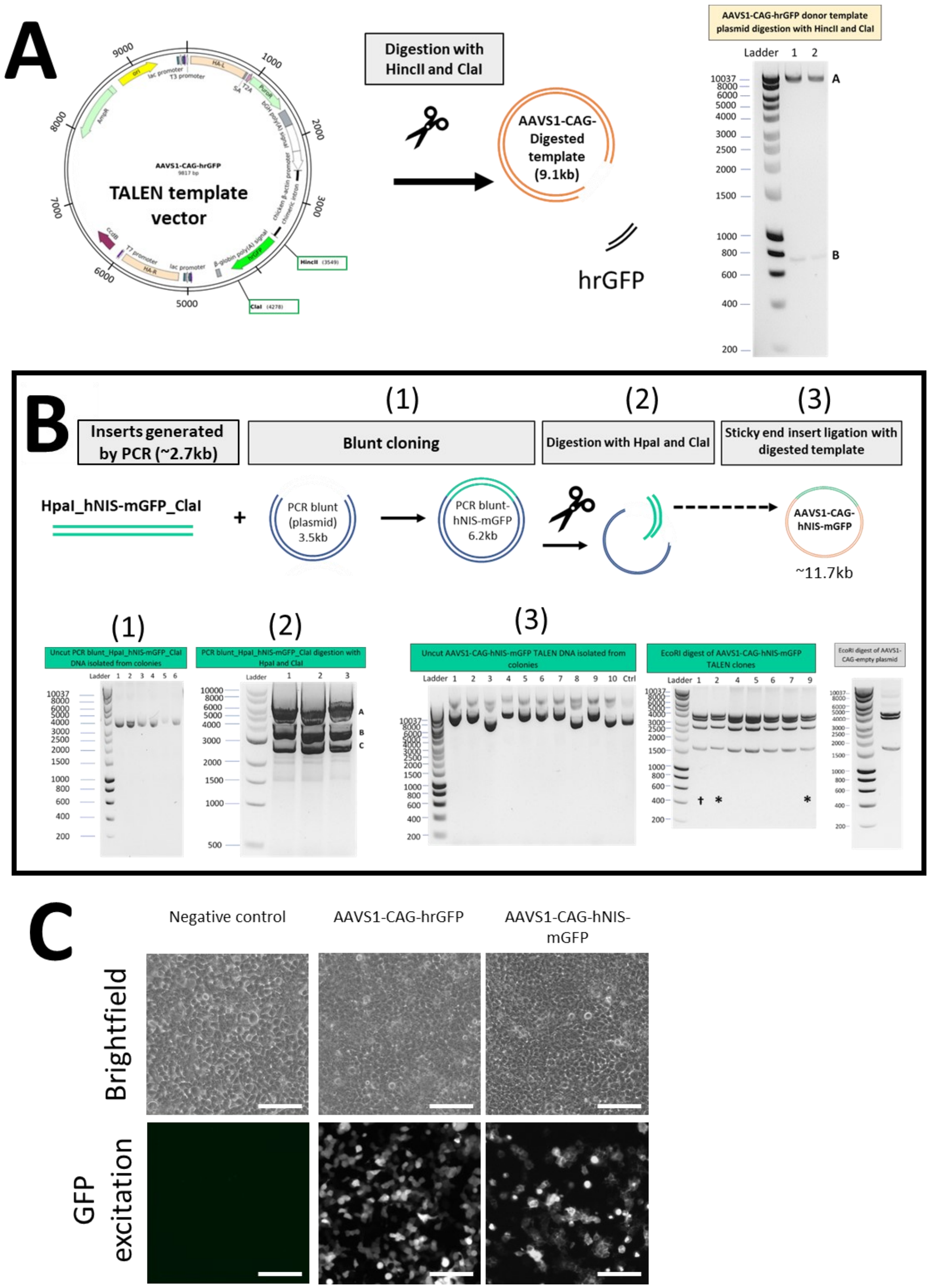


**Figure S1. Generation of the AAVS1-CAG-hNIS-mGFP plasmid. | (A)**The AAVS1-CAG-hrGFP vector was used as starting material and compatible blunt and sticky ends were generated by digestion with HincII and ClaI respectively, products were separated on a 1%

TAE gel. The expected bands of 9.1kb and 700bp are seen, indicated by A and B respectively. DNA from A was isolated for future ligation. **(B)** The blunt end HpaI_hNIS-mGFP_ClaI insert was generated by high-fidelity PCR from primers intended to introduce compatible restriction ends using the template plasmid pLNT_sffv>hNIS-eGFP(A206K). The product was ligated into the PCR blunt plasmid and Miniprep DNA samples corresponding to colonies from the ligation of PCR Blunt plasmid with HpaI_hNIS-mGFP_ClaI inserts were run uncut on 0.8% TAE gels to select samples for further analysis *(Inset 1)* Supercoiled DNA as is present in the uncut sample runs faster than linear DNA, therefore bands were not expected at 6.2kb (the size of PCR blunt + insert) but lower in the gel, with clones where the PCR blunt plasmid had self-ligated or incorporated incomplete nucleotide fragments expected the furthest along the gel given their anticipated smaller size. *(Inset 2)* Digestion with HpaI and ClaI was performed on a subset of predicted successful clones based on the uncut gel results and run on 0.8% TAE gels. Bands correspond to **A**- linearized plasmid, **B**- PCR blunt plasmid, **C**- HpaI_hNIS-mGFP_ClaI. *(Inset 3)* Left gel: Miniprep DNA samples corresponding to colonies from the ligation of digested AAVS1 donor template with HpaI_hNIS-mGFP_ClaI digested inserts were run uncut on 0.8% TAE gels. Circularised empty TALEN template isolated from a previous experiment was run in the last lane of the gel as a control, expected to run further through the gel than successful clones. *(Inset Middle gel)* Plasmid DNA isolated from successfully grown clones was verified by analytical digestion and sanger sequencing. Digestion with EcoRI was performed on the indicatively successful clones based on the uncut gel results and run on 0.8% TAE gels. The expected bands for successful insertion (~1600bp, 2700bp, 3500bp and 3900bp) are seen. Clones with * and † were Sanger sequenced, (*) denotes clones where sequencing results confirm the expected sequence. (†) indicates sequencing revealed a nucleotide deletion. *(Right gel)* Empty AAVS1-CAG donor plasmid was also digested with EcoRI as a control, this shows the expected bands (~1600bp, 3500bp and 3900bp). **(C)** To validate that the sequenced TALEN plasmids yielded expression of hNIS-mGFP HEK 293T cells were transiently transfected and live-cell fluorescence microscopy was performed 48-hours later. As positive controls cells were also transfected with AAVS1-CAG-hrGFP. Expected membrane expression of GFP was seen in the hNIS-TALEN transfected cells indicating fluorescent protein fusion to hNIS. Comparatively, AAVS1-CAG-hrGFP transfected cells demonstrated cytosolic GFP expression, indicating that the GFP transgene was not anchored to a membrane expressed protein. The same TALEN transgene plasmid clone tested for transient expression and sanger sequenced was used in all subsequent gene editing experiments. Scale bars 100µm.

**Figure S2**

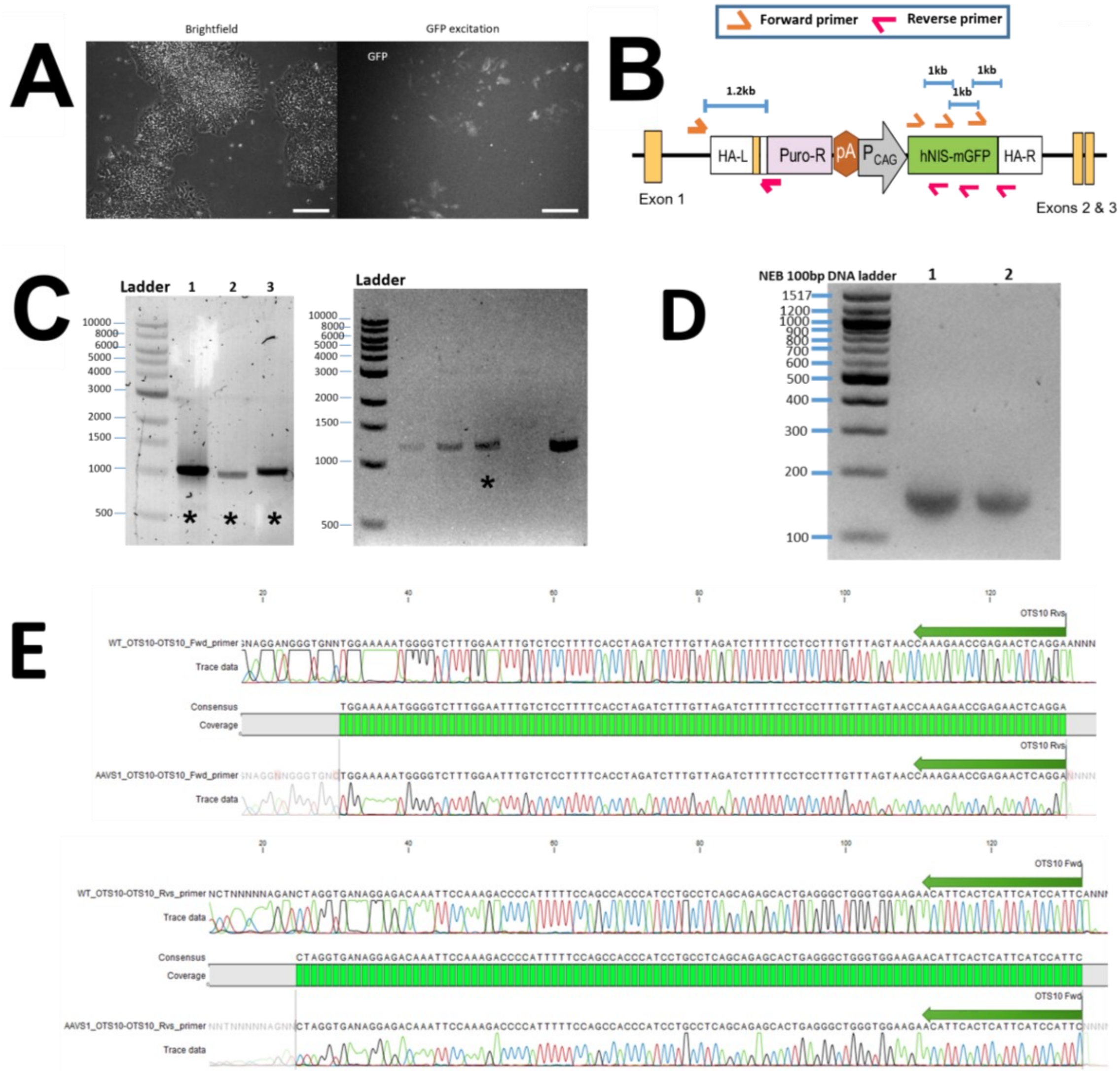


**Figure S2. Analysis of hNIS-mGFP reporter gene incorporation into the *AAVS1* locus of hiPSCs. | (A)** cGMP compliant CGT-RCiB-10 hiPSCs were seeded at low density as small colonies on vitronectin coated 12-well plates. 24-hours after seeding colonies were transfected with 0.4µg each of hAAVS1 TALEN Left and Right plasmids and 1µg of AAVS1-CAG-hNIS-mGFP donor plasmid in Opti-MEM/STEM lipofectamine solution. Media was topped up after 24 hours and live-cell fluorescence microscopy was performed 48-hours after transfection. GFP expression was seen within colonies. Scale bars are 100µm. **(B)** Schematic of expected gene cassette at *PPP1R12C* gene intron 1 (*i.e. AAVS1* locus) following successful gene editing. Half arrows represent regions of primer binding and directionality (orange forward, magenta reverse) and were used for verifying successful integration of the reporter cassette. Expected size of PCR products based on primers pairs are also annotated. **(C)** Genomic DNA was isolated from AAVS1-CAG-hNIS-mGFP hiPSCs and PCR screening reactions performed. PCR products were run 0.8% TAE gels. Left hand gel: products of primer pairs targeting overlapping regions within hNIS-mGFP show the expected 1kb band pattern. Primer pairs were: Lane 1 hNISFwd283/hNISRvs1301, lane 2 hNISFwd837/hNISRvs1821, lane 3 hNISFwd1742/TALENRvs. Right hand gel: Primers targeted within the first PPP1R12C intron and the Puro-R gene in the transgene cassette were used to demonstrate correct integration. Lanes 1-3 show PCR products using genomic AAVS1-CAG-hNIS-mGFP DNA at 25ng, 50ng and 100ng respectively in the PCR reaction. As PCR controls reactions using 100ng of either wildtype unedited CGT-RCiB-10 hiPSC genomic DNA (lane 4, negative control) or H9-AAVS1-CAG-hRGFP hESC genomic DNA (lane 5, positive control). Successful reactions show the

expected 1.2kb band. (*) indicates DNA from these reactions were isolated and the expected results confirmed by Sanger sequencing. **(D)** To determine if any off-target integration occurred the previously published off-target site 10 (OTS10) was genotyped by PCR and sanger sequencing. PCR products from reactions with total genomic showed the expected band pattern (~167bp).Lane 1 = Parental CGT-RCiB-10, Lane 2 = CGT-RCiB-10-AAVS1-CAG-hNIS-mGFP. **(E)** Purified DNA fragments from (D) were Sanger sequenced. Alignment analysis of sequencing trace data showed no difference in Parental and AAVS1-edited gene sequences indicating no off-target integration at OTS10 and no mutations/indels in the edited cells.

**Figure S3**

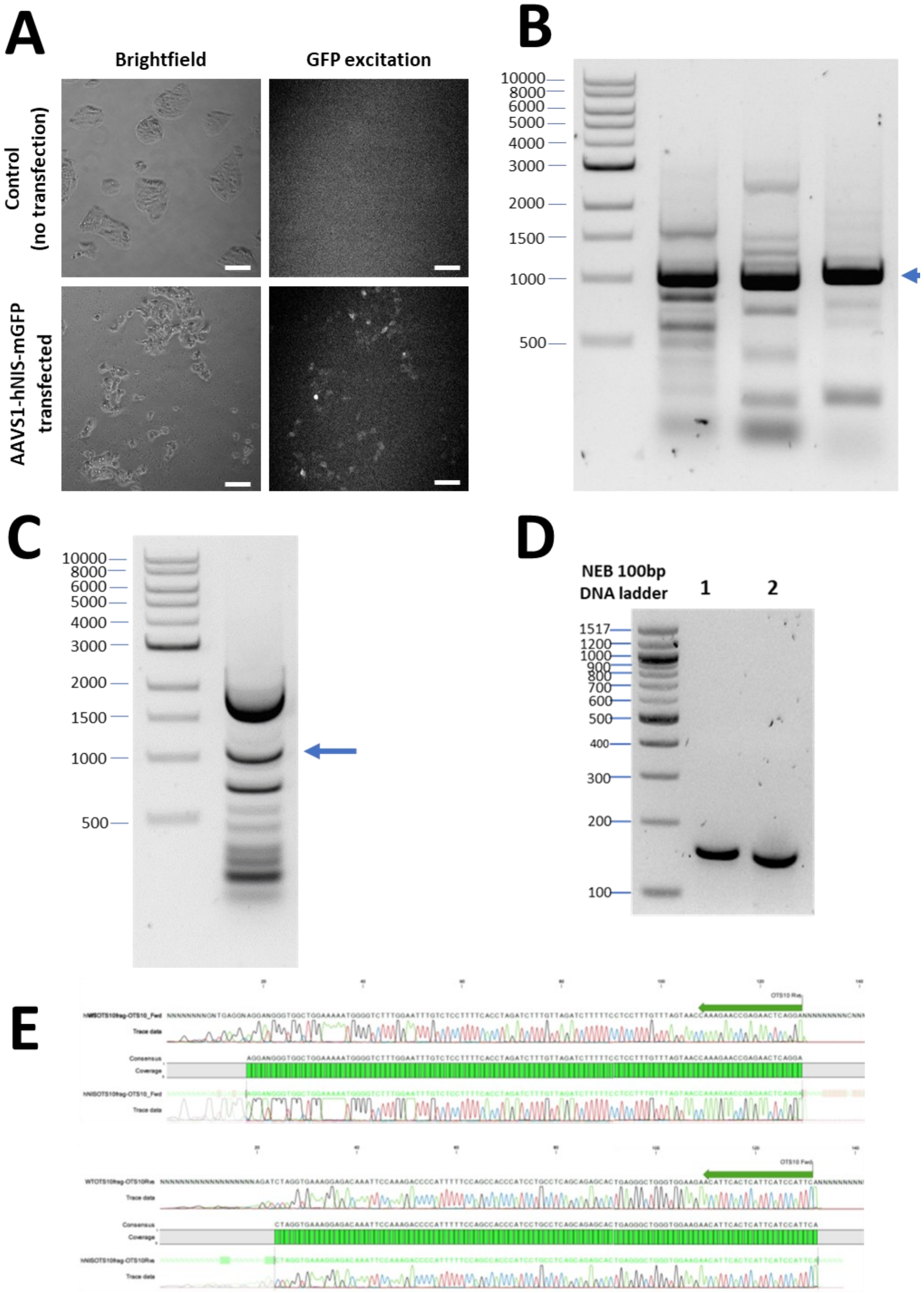


**Figure S3. Analysis of hNIS-mGFP reporter gene incorporation into the AAVS1 locus of HepG2 cells. | (A)** HepG2 cells were seeded at low density in 12-well plates. 24-hours after seeding cells were transfected with 0.4µg each of hAAVS1 TALEN Left and Right plasmids and 1µg of AAVS1-CAG-hNIS-mGFP donor plasmid in Opti-MEM/lipofectamine 2000 solution. Media was topped up after 24 hours and live-cell fluorescence microscopy was performed 48-hours after transfection. Representative fluorescent micrographs demonstrate GFP expression seen within cell clusters. Scale bars 100µm. **(B)** To verify the puromycin selected

polyclonal line demonstrated successful incorporation of the reporter cassette within the AAVS1 locus genomic DNA was isolated from AAVS1-CAG-hNIS-mGFP HepG2s and PCR screening reactions performed. Schematic of expected gene cassette at *PPP1R12C* gene intron 1 (*i.e. AAVS1* locus) following successful gene editing is the same as in Figure S2. PCR products were run 0.8% TAE gels. Blue arrows indicate DNA from these bands were isolated and the expected results confirmed by Sanger sequencing. Products of primer pairs targeting overlapping regions within hNIS-mGFP show the expected 1kb band pattern. Primer pairs were: Lane 1 hNISFwd283/hNISRvs1301, lane 2 hNISFwd837/hNISRvs1821, lane 3 hNISFwd1742/TALENRvs. **(C)** To demonstrate correct integration primers targeted within the first PPP1R12C intron and the Puro-R gene in the transgene cassette were used with genomic AAVS1-CAG-hNIS-mGFP DNA at 100ng used as the template for the PCR reaction. The successful reaction shows the expected ~1.2kb band (which was confirmed by Sanger sequencing). **(D)** To determine if any off-target integration occurred the previously published off-target site 10 (OTS10) was genotyped by PCR and sanger sequencing. PCR products from reactions with total genomic showed the expected band pattern (~167bp). Lane 1 = Parental HepG2, Lane 2 = HepG2-AAVS1-CAG-hNIS-mGFP. **(E)** Alignment analysis of sanger sequencing trace data from the purified DNA fragments showed no difference in Parental and AAVS1-edited gene sequences indicating no off-target integration at OTS10 and no mutations/indels in the edited cells.

**Figure S4**

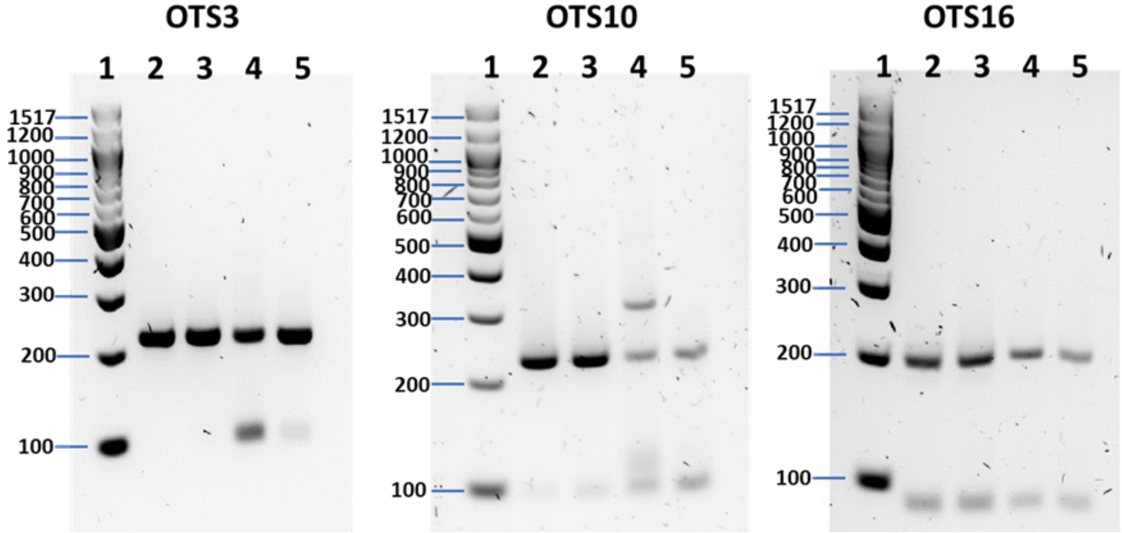


**Figure S4. Off-target site analysis by targeted deep sequencing. |** To determine the presence of any rare off-target indels the previously published off-target sites OTS3, OTS10 and OTS16 were genotyped by PCR, bands of the expected size (OTS3: 221bp, OTS10: 232bp, OTS16: 201bp) were extracted from gels. DNA isolated from extracted gel bands was subject to next generation sequencing (targeted deep sequencing) with sequences compared to the human genome reference sequence to assess indel abundance. In all gels lane 1 = 100bp DNA ladder (DNA), lane 2 = CGT10 hiPSC parental, lane 3 = CGT10-hNIS-mGFP hiPSC, lane 4 = HepG2 parental, lane 5 = AAVS1-CAG-hNIS-mGFP HepG2.

**Figure S5**

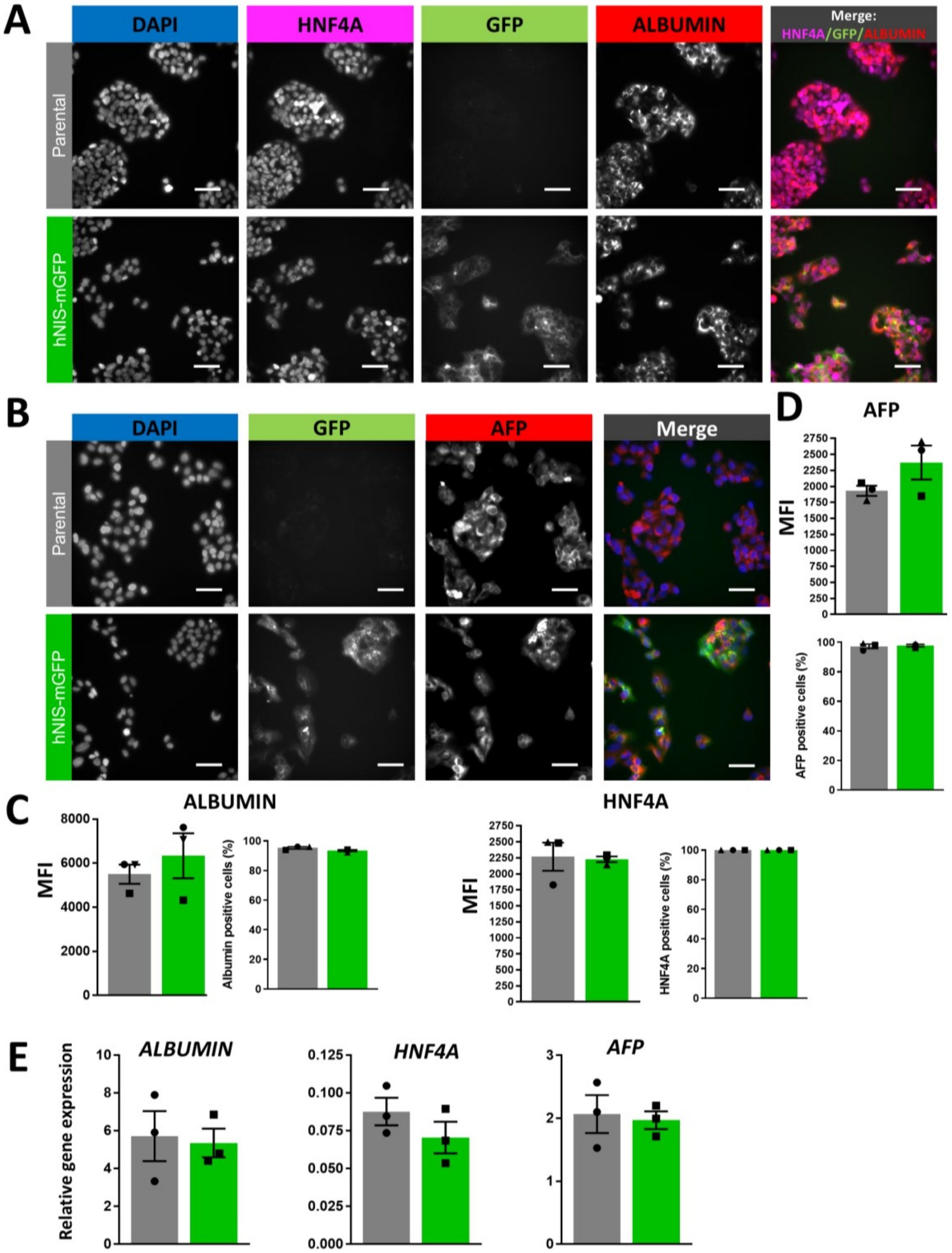


**Figure S5. Verification of retained hepatic phenotype in AAVS1-CAG-hNIS-mGFP HepG2 cells. |** Reporter and parental cells were characterised in parallel to assess the impact of reporter expression on the hepatic phenotype of HepG2 cells. **(A-B)** HepG2 cells were fixed for immunostaining and stained for the hepatocyte markers albumin, HNF4A **(A)** and AFP **(B)**. Cells were imaged with a PerkinElmer Operetta automated high content microscope. Representative fluorescence micrographs from N=3 independent experiments are shown. Quantification of mean fluorescence intensity (MFI) and % positively stained cells for albumin, HNF4A **(C)** and AFP **(D)** was performed using Harmony software V.48 on n>2000 cells per well analysed (triplicate wells per independent experiment), data point symbols (triangle, square, circle) represent independent passages. Error bars SEM. Scale bars 50μm. **(E)** To

confirm IF results total RNA was extracted from HepG2 cells and mRNA expression examined by qPCR. The ΔCt method was used for quantification with sample threshold cycle (Ct) values normalized to the housekeeping gene β-Actin (*ACTB*). N=3 independent experiments (triplicate technical replicates per experiment). Error bars SEM. Student's t-test analysis showed no difference in mean MFI, % of positively stained cells or gene expression for any of the hepatic markers examined (p>0.05).

## Figure S6

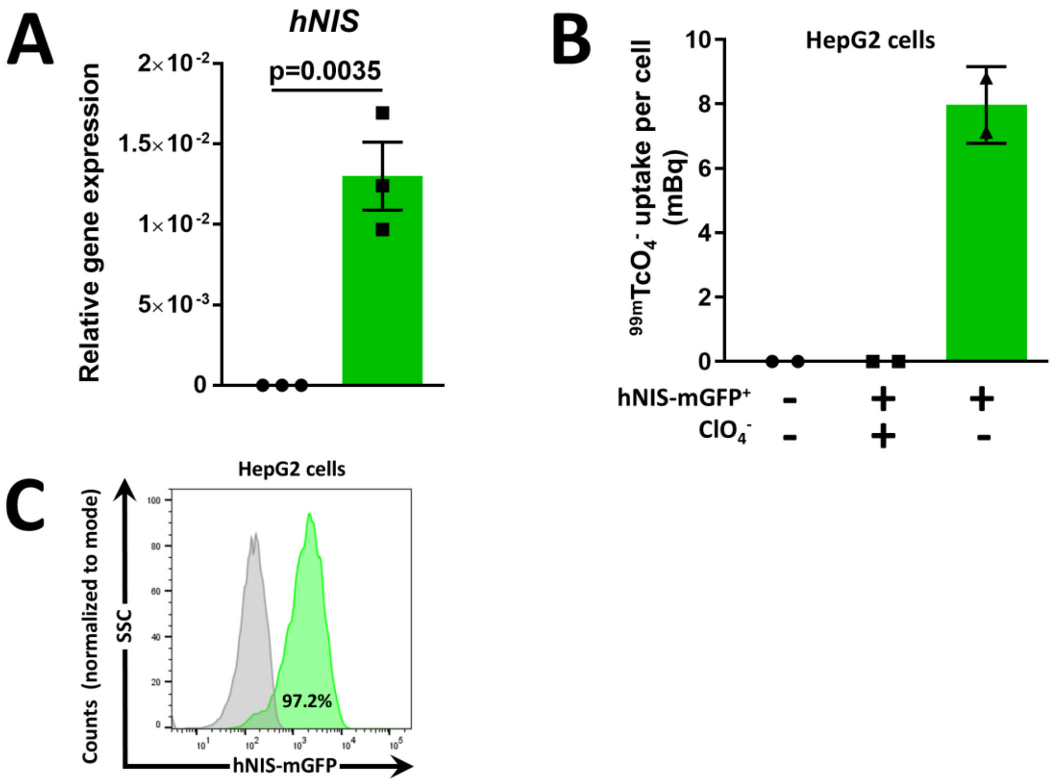


**Figure S6. Verification of hNIS-mGFP expression and function in gene edited HepG2 cells. | (A)** mRNA expression of hNIS in HepG2s was examined by qPCR. Quantification was by the ΔCt method with sample threshold cycle (Ct) values normalized to the housekeeping gene β-Actin. N=3 independent passages were analysed (quadruplicate technical replicates per biological sample). Error bars are SD. Student's t-test shows a significant mean difference in expression. **(B)** hNIS-mGFP function quantified by $^{99m}TcO_4^-$ uptake is shown relative to wild-type cells. The hNIS co-substrate perchlorate served as a $^{99m}TcO_4^-$ uptake specificity control. N=2 biological replicates (triplicate technical replicates per biological sample). **(C)** Flow cytometry was used to examine hNIS-mGFP expression relative to wild-type HepG2 cells. Representative histograms are shown (N=3 independent experiments).

**Figure S7**

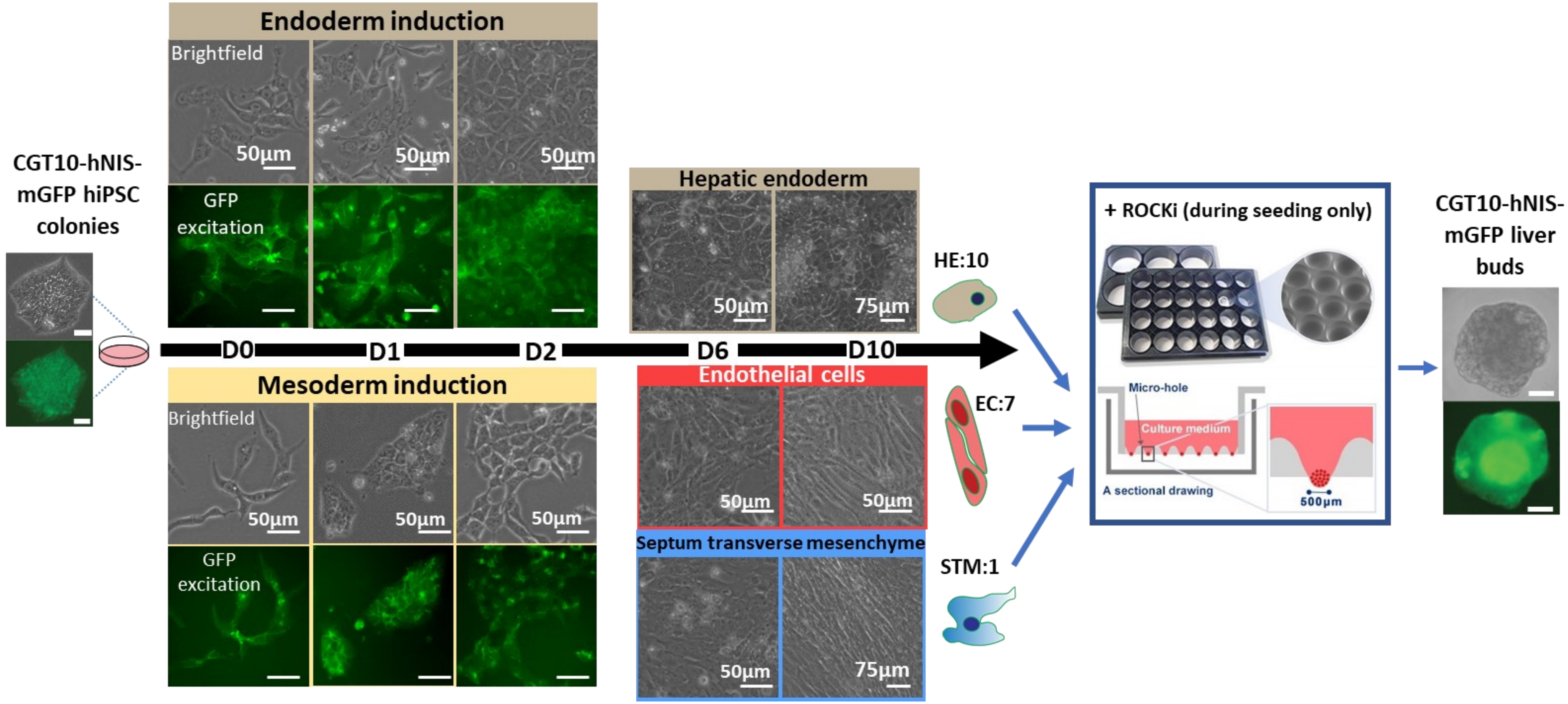


**Figure S7. Graphical summary of co-culture platform to yield in vivo trackable CGT10-hNIS-mGFP hiPSC derived LBs. |** This summary graphic depicts the process of progressing CGT10-hNIS-mGFP hiPSCs to CGT10-hNIS-mGFP LBs. hiPSCs are differentiated into the three progenitor populations hepatic endoderm (HE), endothelial cells (EC) and septum transverse mesenchyme (STM) and combined with a cell number ratio of 10:7:1 in Elplasia microwell plates (with the addition of ROCKi to culture media during seeding only). Cells self-condense overnight to produce liver organoids within each microwell. Micrograph scale bars 50μm unless otherwise indicated. As seen with previous differentiation protocols differentiated progenitor cells retain expression of the hNIS-mGFP reporter. The microwell plate image included in this schematic was taken from the manufacturer brochure/protocol.

**Figure S8**

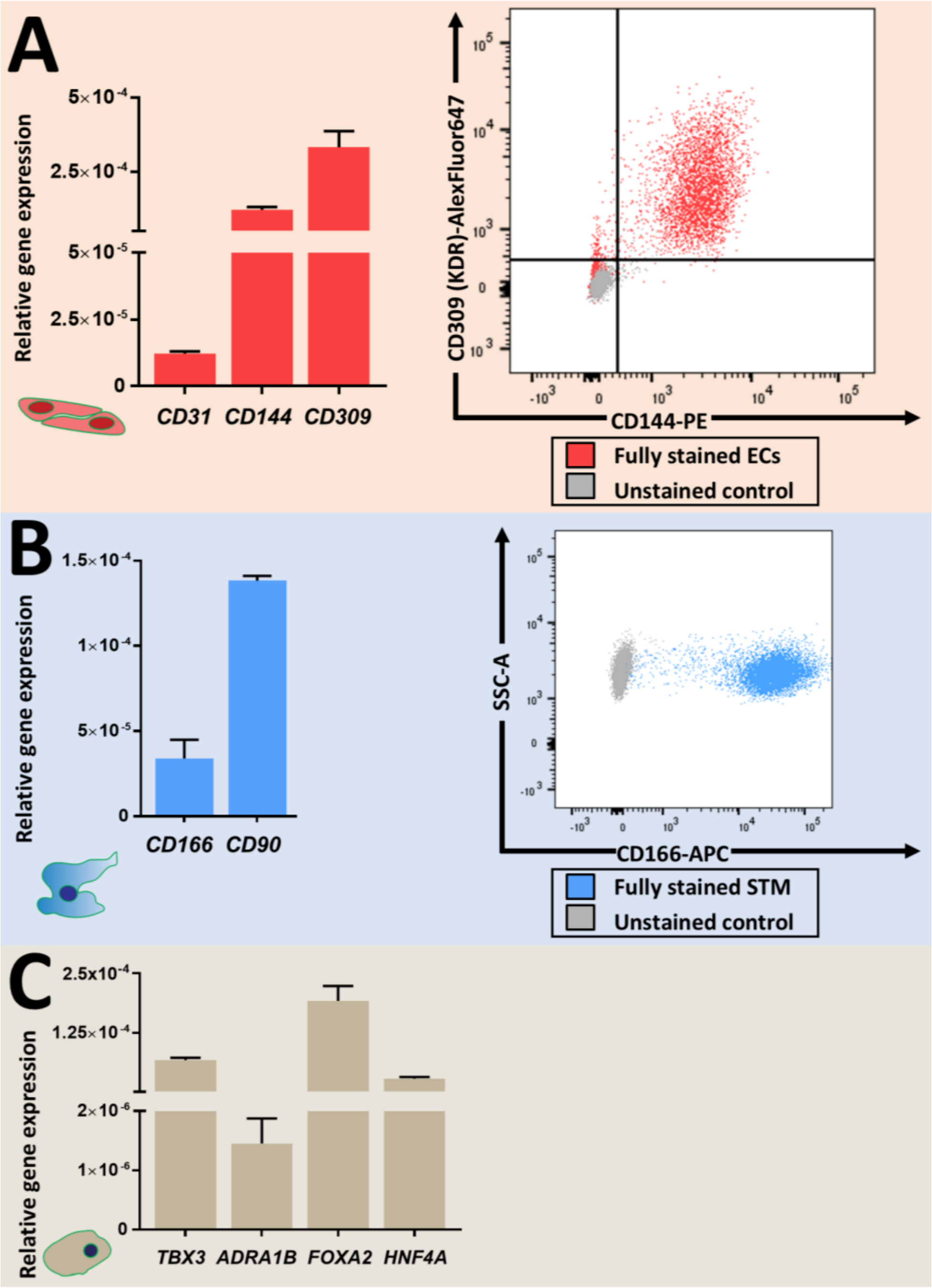


**Figure S8. Confirmation of differentiated cell lineages at D10. |** To verify differentiation yielded the expected progenitor cell populations phenotyping via flow cytometry and qPCR were performed. Results show either representative flowcytometry scatter plots (N=2 independent differentiations) or qPCR results (N=3 independent differentiations, duplicate technical replicates, error bars are SEM) expressed relative to the housekeeping gene *18S rRNA*. **(A)** Scatter plots showing overlaid unstained control ECs (grey) and ECs stained for the endothelial markers CD309 and CD144. Results confirm the presence of differentiated ECs co-expressing these markers and are verified by mRNA expression. **(B)** Overlaid scatter plots with unstained control STM (grey) and STM stained for the CD90 mesenchyme marker (blue) demonstrating the presence of differentiated STM. Results are confirmed by mRNA expression. **(C)** mRNA expression confirming presence of a hepatic endoderm population through analysis of the markers: *TBX3*, adrenoceptor α1B (*ADRA1B*), *HNF4α* and *FOXA2*.

**Figure S9**

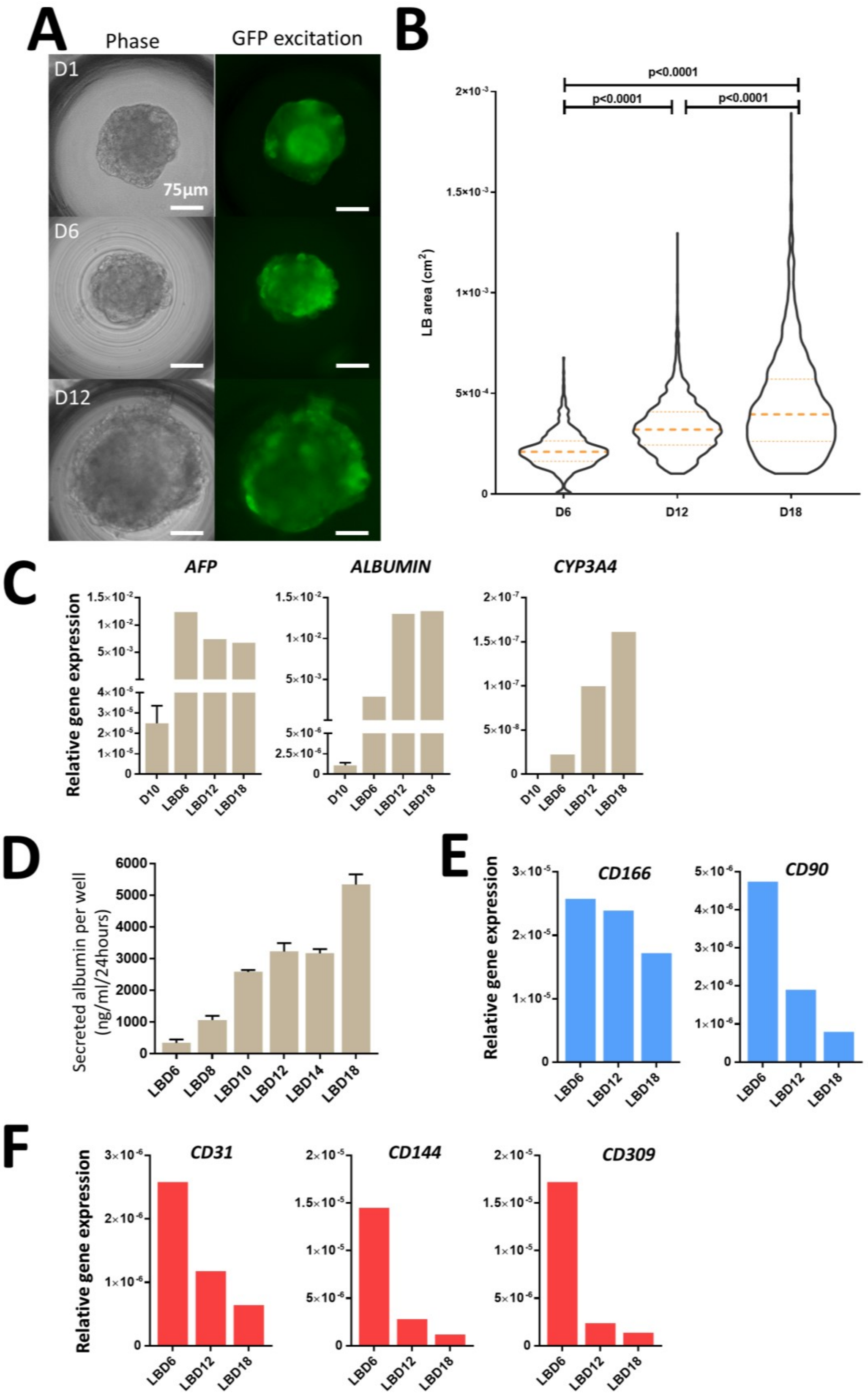


**Figure S9. Characterisation of LB morphology and retained multilineage populations over time. |** To confirm LB maturation over time, and retention of the three cell populations total RNA was extracted from LBs following different periods of maturation *in vitro* and mRNA expression examined by qPCR. The ΔΔCt method was used for quantification with sample

threshold cycle (Ct) values normalized to the housekeeper 18srRNA. **(A)** Representative live cell micrographs of cultured LBs showing change in LB morphology over time, from an initially tight packed structure which relaxes gradually over time in line with bud expansion in culture. **(B)** Violin plot demonstrating the frequency distribution of LB area over time. One Way ANOVA with Tukey's multiple comparison correction of mean area at each timepoint shows a significant difference between each timepoint. Results are pooled data from n=3 (D6) or n=4 wells (D12 and D18) corresponding to >600 LBs per timepoint. Orange dotted lines represent median and quartiles. **(C)** Markers of mature hepatocytes were examined over time and compared to hepatic endoderm (HE) gene expression. HE N=3 biological replicates, duplicate technical replicates. LBs N=1 biological replicate/well per timepoint, duplicate technical replicates. **(D)** To verify albumin qPCR results culture media was collected throughout LB maturation and secreted albumin concentration determined by ELISA. Results shown are triplicate wells of LBs per timepoint, duplicate technical replicates. **(E)** Mesenchyme and **(F)** Endothelial marker expression examined over time to confirm retained presence of these cell populations within the LBs N=1 biological replicate/well per timepoint, duplicate technical replicates.

**Figure S10**

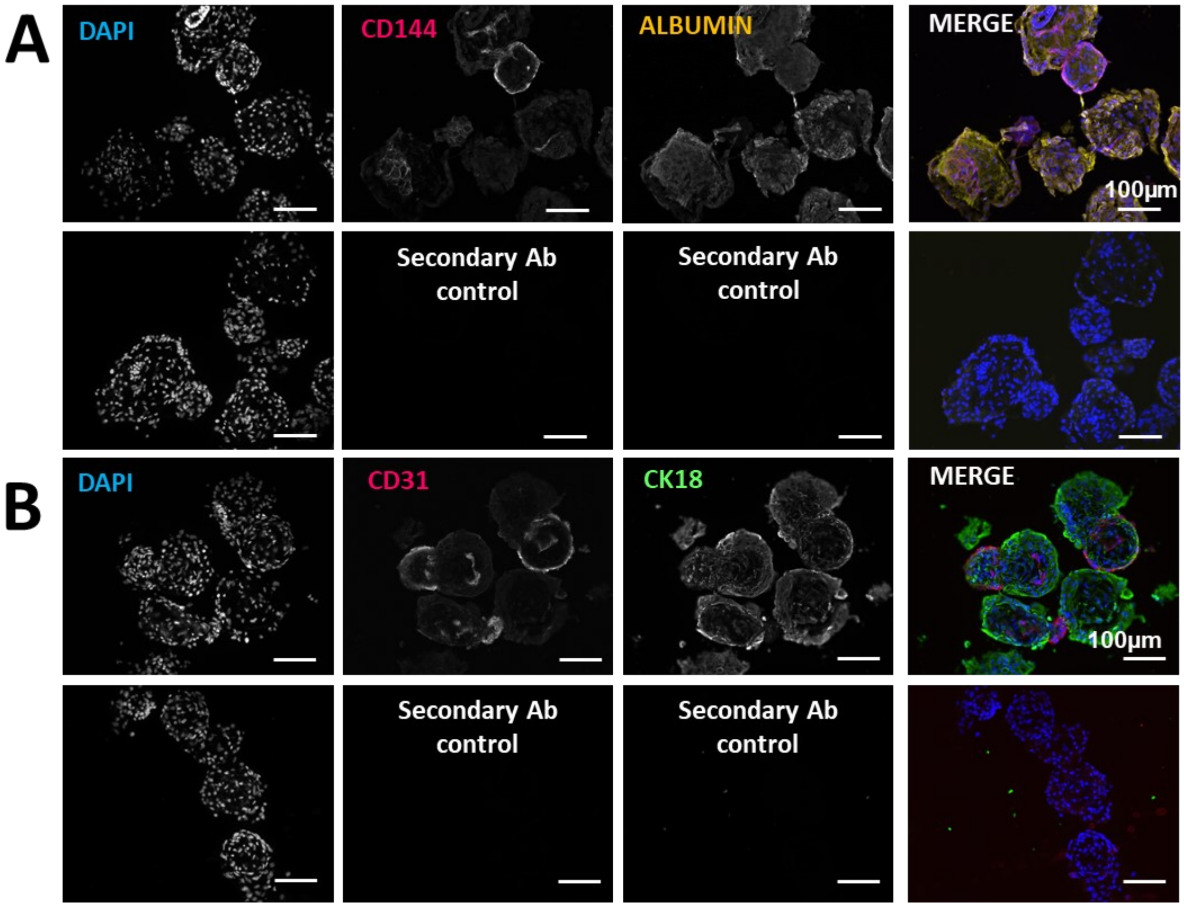


**Figure S10. Immunostaining of LBs following 11 days of in vitro co-culture. |** LBs cultured *in vitro* were fixed at 11 days after self-condensation. Immunostaining using human antigen targeted antibodies for mature vascular (CD144, CD31) and hepatic markers (Albumin, CK18) was performed **(A)** and **(B)**. LBs show positive staining for all markers, indicating the retained presence of human endothelial and hepatic cell populations. An immature vascular network is seen, with some clusters of hepatic cells (albumin or CK18 positively stained) visibly surrounded by endothelial cells (CD144 or CD31 positive) whereas in other LBs hepatic cells encapsulating the developing vascular network.

Figure S11

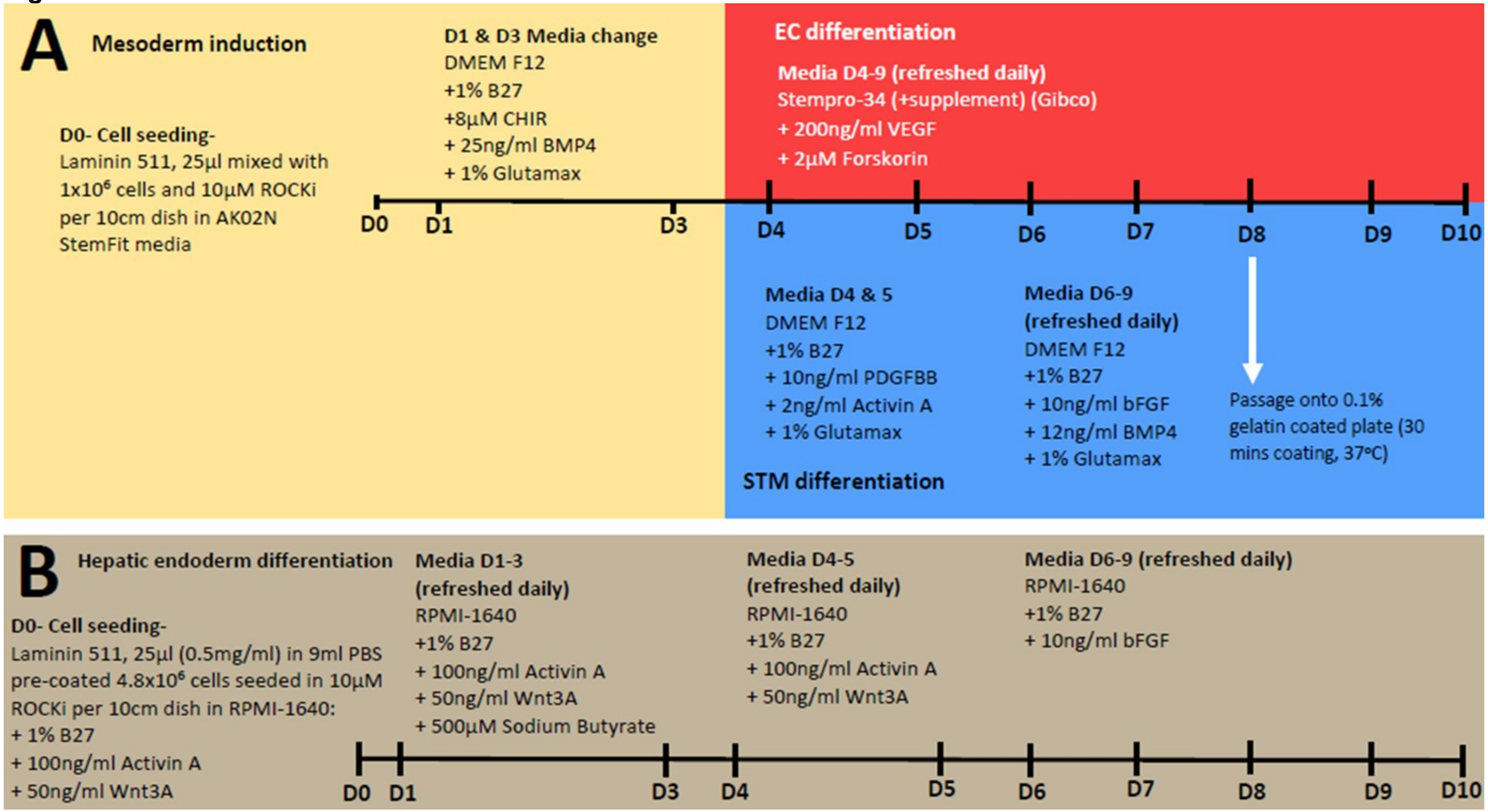


**Figure S11. Synopsis of iPSC differentiation protocol for LB progenitor populations | (A)** Representation of mesoderm induction process and subsequent divergence in protocol to produce Endothelial cell (EC)s or septum transverse mesenchyme (STM) **(B)** Representation of Hepatic endoderm differentiation process.

**Figure S12**

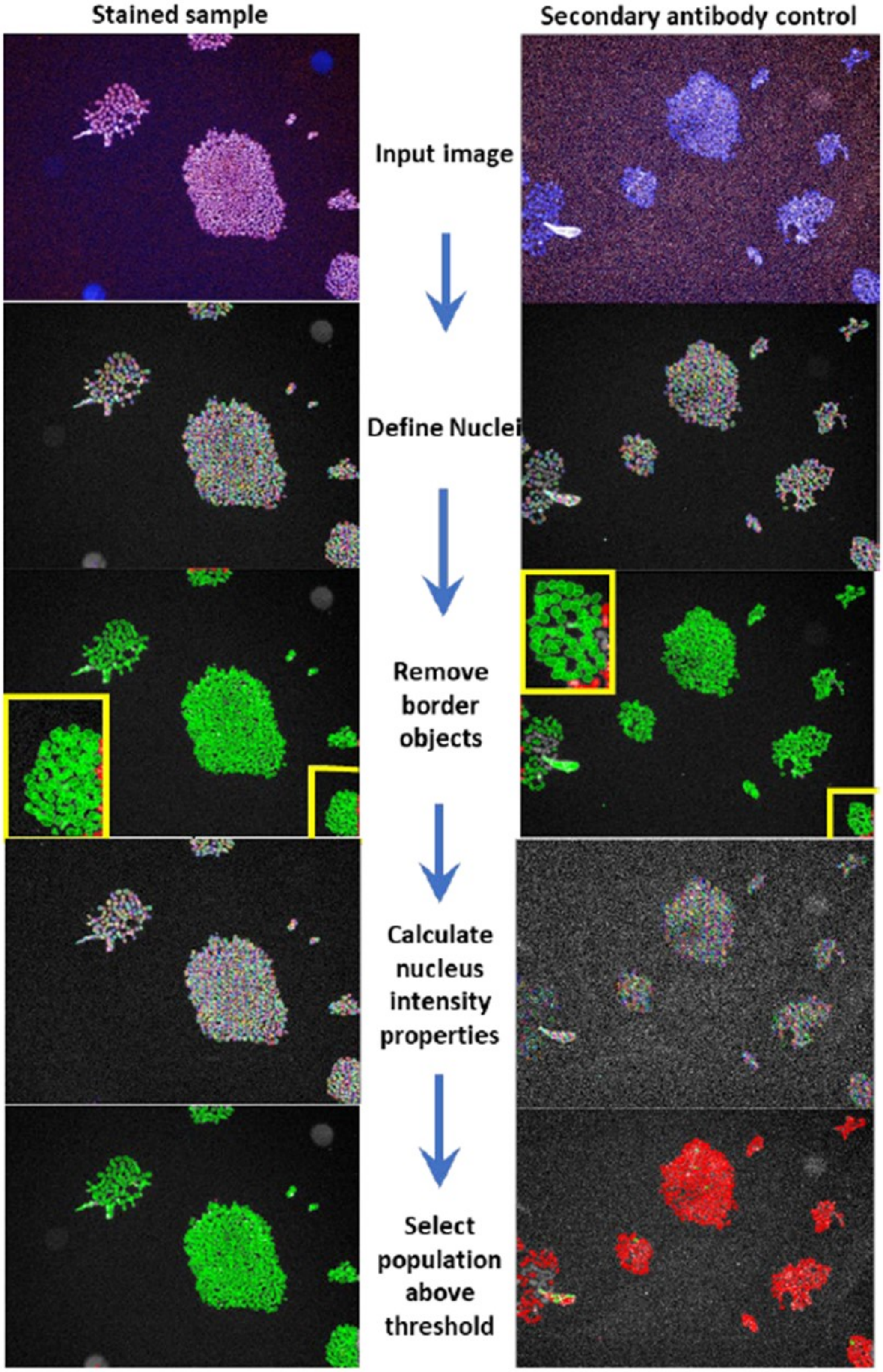


**Figure S12. Workflow example from Harmony analysis pipeline of nuclear stained hiPSCs. |** Nuclei were segmented in the DAPI channel of input images using the inbuilt 'M' Method. The M method is optimal for segmenting nuclei in close proximity providing nuclei are typically of homogenous size[1] as demonstrated in throughput imaging studies of hiPSCs previously[2]. The segmented region is defined as 'Nuclei'. Cells on border regions of the image

field of view were excluded from analyses, with the refined population defined as ‘Nuclei selected’. The threshold mean fluorescence intensity (MFI) for positively stained ‘Nuclei selected’ populations was set based on background intensity in the ‘Nuclei Selected’ population of immunostaining control wells analysed, thus for each independent biological experiment positive cells had values greater than the nuclear MFI of the secondary control well +1.5 standard deviations in the appropriate fluorescence channel. A minimum of 15 fields of view per well were analysed. To calculate the proportion of positively stained nuclei the following calculation was applied: ([Number of objects: Nuclei selected MFI> (threshold value)]/Number of objects nuclei selected)*100

**Figure S13**

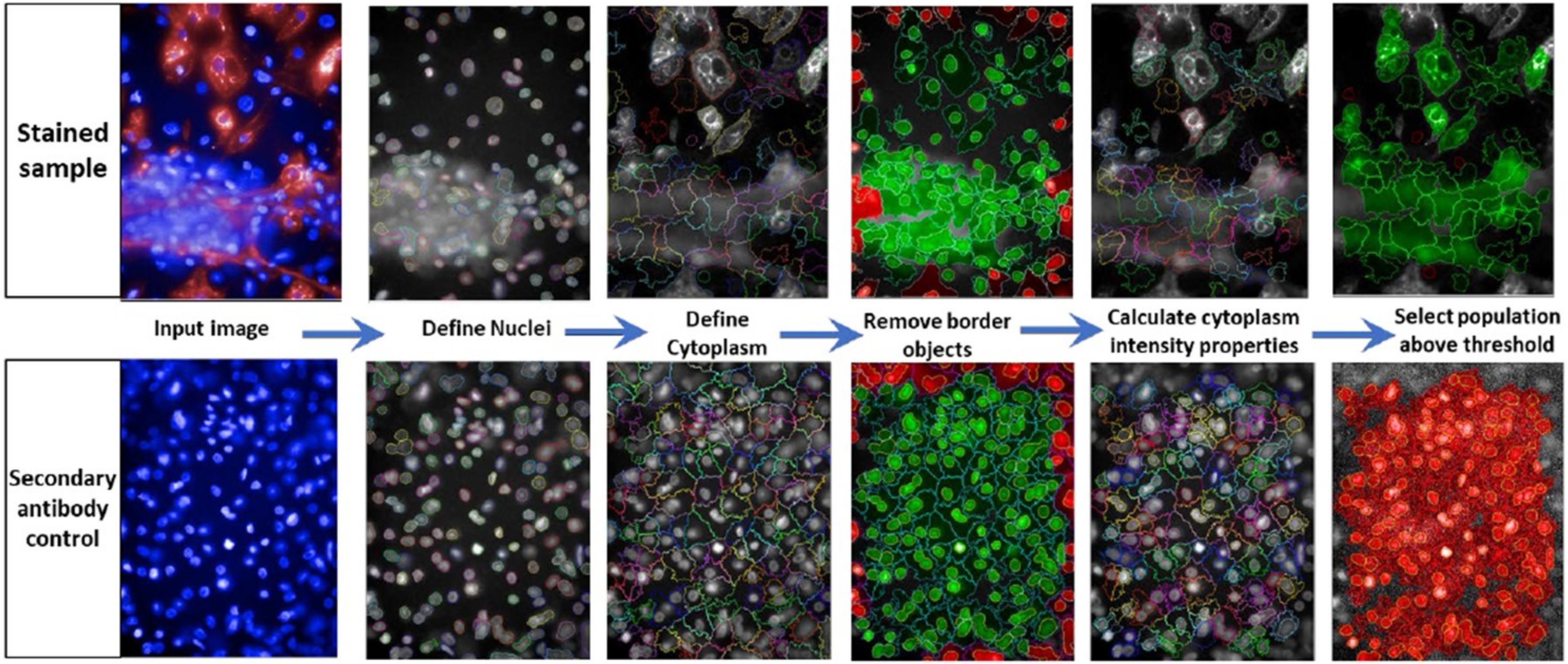


**Figure S13. Workflow example from Harmony analysis pipeline of cytoplasmic stained HLCs. |** The cytoplasmic staining pipeline was defined comparably to the nuclear staining analysis pipeline, except that after defining Nuclei the cytoplasmic region was defined using method B. This is effective in micrographs where not all cytoplasmic regions are stained but the nuclei are identified in another channel, thereby allowing examination of populations which include cells negative for the cytoplasmic protein of interest. Cytoplasmic regions were defined initially using Harmony in-built algorithms to detect regions in the image with higher intensity than the background using the most common parameters for the fluorochrome, segmentation, then optimised by modifying the intensity of signal corresponding to a cell to improve the determination of the cytoplasmic region[1]. Border cells were excluded from the pipeline leaving a 'Cells selected' population. MFI was calculated for the cytoplasmic region of the Cells selected population using the appropriate fluorescence channel for the secondary antibody fluorochrome (nucleus region excluded). Thresholds for positive staining were set using the same protocol as for nuclear staining (positively stained cells are those with MFI > [cytoplasmic MFI of the secondary control well + 1.5SD]).

## SUPPLEMENTAL TABLES

### Table S1. Primer sequences used for analytical PCR and Sanger Sequencing.

| Primer name | Sequence (5' to 3') |
|---|---|
| **DNA803/AAVS1 locus primer** | TCGACTTCCCCTCTTCCGATG |
| **DNA804/transgene targeted primer** | GAGCCTAGGGCCGGGATTCTC |
| **hNIS FWD 283** | CTTCTGAACTCGGTCCTCAC |
| **hNISREV1301** | GCAGGCCGGCAGGAACATTC |
| **hNISFWD837** | GCTGGCCCTGCTCATCAA |
| **hNISREV1821** | AAAAACAGACGATCCTCATTG |
| **hNISFWD1742** | CCATCCTGGATGACAACTTGG |
| **TALENREV** | TTTTGGCAGAGGGAAAAAGA |
| **OTS10 FWD** | GAATGGATGAATGAGTGAATG |
| **OTS10 RVS** | TCCTGAGTTCTCGGTTCTTTG |

### Table S2. Primer sequences used for targeted deep sequencing. 
Illumina adaptor sequences are underlined.

| Primer name | Sequence (5' to 3') |
|---|---|
| **OTS3 FWD** | ACACTCTTTCCCTACACGACGCTCTTCCGATCTTGAGCCATCATGCCCAG |
| **OTS3 RVS** | GACTGGAGTTCAGACGTGTGCTCTTCCGATCTCCTTAAAGCTTTGTACACAAATTCA |
| **OTS10 FWD** | ACACTCTTTCCCTACACGACGCTCTTCCGATCTGAATGGATGAATGAGTGAATGT |
| **OTS10 RVS** | GACTGGAGTTCAGACGTGTGCTCTTCCGATCTTCCTGAGTTCTCGGTTCTTTG |
| **OTS16 FWD** | ACACTCTTTCCCTACACGACGCTCTTCCGATCTAGCTGTTGCAGGGCTCTC |
| **OTS16 RVS** | GACTGGAGTTCAGACGTGTGCTCTTCCGATCTAATGGCACCAGAGGGGTCT |

## Table S3. PCR thermocycling conditions.

| Step | Temperature (ºC) | Time |
|---|---|---|
| **SYBR Green protocol** | | |
| **Denaturation** | 95 | 3min |
| **Denature** (40 cycles)<br>**Anneal/Extend** | 95<br>60 | 3s<br>20s |
| **Extension** | 60-90 | 0.5ºC/s |
| **SYBR Thunderbird protocol** | | |
| **Denaturation** | 95 | 10min |
| **Denature** (45 cycles)<br>**Anneal/Extend** | 95<br>60 | 15s [4.4º/s]<br>1min [2.2ºC/s] |
| **Cooling** | 40 | 30s |
| **Taq DNA polymerase protocol** | | |
| **Denaturation** | 98 | 3min |
| **Annealing (35 cycles)** | 98<br>92<br>87<br>82<br>77<br>72 | 25s<br>12s<br>12s<br>12s<br>14s<br>1min 40s |
| **Extension** | 72 | 20min |
| **Hold** | 4 | ∞ |
| **Q5 DNA polymerase protocol** | | |
| **Denaturation** | 98 | 30s |
| **Annealing (35 cycles)** | 98<br>92<br>87<br>82<br>77 | 20<br>12<br>12<br>12<br>12 |
| **Extension** | 72 | 90 |
| **Hold** | 4 | ∞ |

## Table S4. Targeted deep sequencing analysis of off-target sites.

| Off-target site | Sample | Total reads | InDels | %InDels | Proportion of InDels relative to parental (%) |
|---|---|---|---|---|---|
| **OTS3** | CGT10 hiPSC (parental) | 58713 | 1321 | 2.25 | / |
| | CGT10.AAVS1-hNIS-mGFP hiPSC | 43365 | 1069 | 2.47 | +0.22 |
| | HepG2 (parental) | 33200 | 972 | 2.93 | / |
| | AAVS1-hNIS-mGFP HepG2 | 63267 | 1274 | 2.01 | -0.92 |
| **OTS10** | CGT10 hiPSC (parental) | 48784 | 2159 | 4.43 | / |
| | CGT10.AAVS1-hNIS-mGFP hiPSC | 46732 | 2730 | 5.84 | +1.41 |
| | HepG2 (parental) | 16446 | 835 | 5.08 | / |
| | AAVS1-hNIS-mGFP HepG2 | 14678 | 828 | 5.64 | +0.56 |
| **OTS16** | CGT10 hiPSC (parental) | 32605 | 284 | 0.87 | / |
| | CGT10.AAVS1-hNIS-mGFP hiPSC | 23694 | 211 | 0.89 | +0.02 |
| | HepG2 (parental) | 25785 | 241 | 0.93 | / |
| | AAVS1-hNIS-mGFP HepG2 | 21413 | 220 | 1.03 | +0.10 |

## Table S5. Quantitative real-time PCR gene specific primers and probes.

| qPCR primers (SYBR Green) for hepatic analysis | | |
|---|---|---|
| **Primer name** | **Primer sequence** | |
| *α-fetoprotein FWD* | TGAATCCAGAACACTGCATAGAA | |
| *α-fetoprotein REV* | TATGGTAGCCAGGTCAGCTA | |
| *Albumin FWD* | CGTCGAGATGCACACAAGA | |
| *Albumin REV* | GATACTGAGCAAAGGCAATCAAC | |
| *Cytokeratin-18 FWD* | TGGTCACCACACAGTCTGCT | |
| *Cytokeratin-18 REV* | CCAAGGCATCACCAAGATTA | |
| *CYP3A4 FWD* | CCTGGTGCTCCTCTATCTATATG | |
| *CYP3A4 REV* | AGCCCTTATGGTAGGACAAA | |
| *FOXA2 FWD* | GAGCGGTGAAGATGGAAG | |
| *FOXA2 REV* | TGTTCATGCCGTTCATCC | |
| *HNF1β FWD* | GCGTCCCAGCAAATCTT | |
| *HNF1β REV* | TTCTGCCCTGTTGCATTC | |
| *HNF4A FWD* | GTGGTGGACAAAGACAAGAG | |
| *HNF4A REV* | TGGACGGCTTCCTTCTT | |
| *TBX3 FWD* | TGACATCTTGAAACTCCCTTATAG | |
| *TBX3 REV* | ACTGGGTTATCTTATCATTCTGG | |
| **qPCR primers (SYBR Thunderbird) for LB analysis** | | |
| **Primer name** | **Primer sequence** | **Roche Probe ID** |
| *Albumin FWD* | AATGTTGCCAAGCTGCTGA | 27 |
| *Albumin REV* | CTTCCCTTCATCCCGAAGTT | 27 |
| *PECAM1/CD31 FWD* | CAGAGAGACCGGCTGTGG | 46 |
| *PECAM1/CD31 REV* | CATTGTTCCCGGTTTCCA | 46 |
| *CD309/KDR FWD* | CGGAAGAACAATGTAGTCTTTGC | 18 |
| *CD309/KDR REV* | GAACATTTGGGAAATCTCTTGC | 18 |
| *hALCAM1/CD166 FWD* | CAGTTCCTGCCGTCTGCT | 34 |
| *hALCAM1/CD166 REV* | CTGAATTTACAGTATACCATCCAAGG | 34 |
| *hCYP3A4 FWD* | GATGGCTCTCATCCCAGACTT | 2 |
| *hCYP3A4 REV* | AGTCCATGTGAATGGGTTCC | 2 |
| *TBX3 FWD* | TCCTCCCTTTGAAGTGCATTA | 16 |
| *TBX3 REV* | GGGGGAAAAAGCAAACAAA | 16 |
| *Cadherin5/CD144/VE-Cadherin FWD* | GCAGTCCAACGGAACAGAA | 30 |
| *Cadherin5/CD144/VE-Cadherin REV* | CATGAGCCTCTGCATCTTCC | 30 |
| *Thy-1/CD90 FWD* | CAGAACGTCACAGTGCTCAGA | 66 |
| *Thy-1/CD90 REV* | GAGGAGGGAGAGGGAGAGC | 66 |
| *AFP FWD* | TCCTTGTAAGTGGCTTCTTGAAC | 61 |
| *AFP REV* | TGTACTGCAGAGATAAGTTTAGCTGAC | 61 |
| *HNF4A FWD* | AGCAACGGACAGATGTGTGA | 27 |
| *HNF4A REV* | TCAGACCCTGAGCCACCT | 27 |
| *FOXA2 FWD* | AGACGGTGTTGCAGAGACG | 7 |
| *FOXA2 REV* | CGGGTGAAGAAGACTGCTG | 7 |
| *ADRA1B FWD* | CCTTCTTCATCGCTCTACCG | 20 |
| *ADRA1B REV* | GGGTTGAGGCAGCTGTTG | 20 |
| *CK18 FWD* | GCTCTGGTTCCCGGATCT | 22 |
| *CK18 REV* | TCTGGATGCCTCCCATTC | 22 |

## SUPPLEMENTAL REFERENCES


1. Perkin Elmer. *Operetta Operation Manual*. (2013).
2. Leha, A. et al. A high-content platform to characterise human induced pluripotent stem cell lines. Methods **96**, 85 (2016).
